# *In vitro* characterization of 3D culture-based differentiation of human liver stem cells

**DOI:** 10.3389/fcell.2024.1352013

**Published:** 2024-02-08

**Authors:** Marta Tapparo, Gabriele Saccu, Chiara Pasquino, Valentina Fonsato, Claudio Medana, Valentina Schiavo, Enrica Mecarelli, Monica Maccagno, Lorenzo Silengo, Stefania Bruno, Giovanni Camussi, Maria Beatriz Herrera Sanchez

**Affiliations:** ^1^ Department of Medical Sciences, University of Torino, Turin, Italy; ^2^ Molecular Biotechnology Centre, University of Torino, Turin, Italy; ^3^ Department of Molecular Biotechnology and Health Sciences, Turin, Italy; ^4^ Officina Farmaceutica, University of Torino, Turin, Italy; ^5^ 2i3T, Società per la Gestione dell’incubatore di Imprese e per il Trasferimento Tecnologico, University of Torino, Turin, Italy

**Keywords:** differentiation, human liver stem cells, rotary cell culture system, Indocyanine green, urea cycle

## Abstract

**Introduction:** The lack of functional hepatocytes poses a significant challenge for drug safety testing and therapeutic applications due to the inability of mature hepatocytes to expand and their tendency to lose functionality *in vitro*. Previous studies have demonstrated the potential of Human Liver Stem Cells (HLSCs) to differentiate into hepatocyte-like cells within an *in vitro* rotary cell culture system, guided by a combination of growth factors and molecules known to regulate hepatocyte maturation. In this study, we employed a matrix multi-assay approach to comprehensively characterize HLSC differentiation.

**Methods:** We evaluated the expression of hepatic markers using qRT-PCR, immunofluorescence, and Western blot analysis. Additionally, we measured urea and FVIII secretion into the supernatant and developed an updated indocyanine green *in vitro* assay to assess hepatocyte functionality.

**Results:** Molecular analyses of differentiated HLSC aggregates revealed significant upregulation of hepatic genes, including CYP450, urea cycle enzymes, and uptake transporters exclusively expressed on the sinusoidal side of mature hepatocytes, evident as early as 1 day post-differentiation. Interestingly, HLSCs transiently upregulated stem cell markers during differentiation, followed by downregulation after 7 days. Furthermore, differentiated aggregates demonstrated the ability to release urea and FVIII into the supernatant as early as the first 24 h, with accumulation over time.

**Discussion:** These findings suggest that a 3D rotation culture system may facilitate rapid hepatic differentiation of HLSCs. Despite the limitations of this rotary culture system, its unique advantages hold promise for characterizing HLSC GMP batches for clinical applications.

## 1 Introduction

Liver transplantation is the main reliable treatment for patients with end-stage liver disease (Zarrinpar and Busuttil, 2013). However, the shortage of available organs, high cost, risk of rejection, and need for immunosuppression have increased in developing alternative approaches, such as cell therapy (Vacanti and Kulig, 2014). Hepatocytes are the primary source of cells investigated for cell therapy (Gramignoli et al., 2015). However, several limitations hinder the *in vivo* use, including low *in vitro* expansion ability, rapid loss of function, and substantially reduced vitality after thawing from cryostorage. Additionally, it is difficult to obtain a sufficient number of hepatocytes for human treatment due to limited access to human liver tissue.

In recent years, researchers have investigated the therapeutic potential of stem cells as an alternative source of mature hepatocytes (Tsolaki and Yannaki, 2015).

Various stem cell sources have been considered, each with its own advantages and disadvantages. For example, embryonic stem cells (ESCs) are a promising source, but their use is restricted due to ethical concerns (Li et al., 2021). An alternative is Induced Pluripotent Stem Cells (iPSCs), which can be easily obtained from autologous differentiated cells, such as dermal fibroblast, through cell reprogramming. However, iPSCs are expensive and have safety concerns that limit their widespread clinical use (Thanaskody et al., 2022).

Mesenchymal stromal cells (MSCs) are adult stem cells that have been widely used in different applications (Han et al., 2019). They can be easily obtained from various tissues and expanded *in vitro* (Gramignoli et al., 2015). MSCs also maintain their plasticity *in vitro* for several passages with few karyotype modifications and can differentiate into hepatocyte-like cells under specific culture conditions (Berebichez-Fridman and Montero-Olvera, 2018; Zhang et al., 2022).

Human Liver Stem Cells (HLSCs) are a resident stem cell population in the liver with high *in vitro* self-renewal capabilities (Herrera et al., 2006). HLSCs display hepatic commitment, expressing human albumin, alpha-fetoprotein (αFP), and variable levels of cytokeratin (CK) 8 and CK18. They also express markers of pluripotency, such as Nanog, OCT3/4, Sox2 and Musashi (Herrera et al., 2013). Similar to MSCs, HLSCs are positive for CD90, CD105, CD29, CD73 and CD44 and lack CD34, CD14 and CD45 expressions (Herrera et al., 2006).

HLSCs have immunomodulatory abilities that may prevent rejection during transplantation (Bruno et al., 2016). They do not express Human Leukocyte Antigens (HLA) class II or costimulatory molecules such as CD40, CD80, and CD86. Additionally, HLSCs inhibit T cell proliferation and dendritic cell differentiation, similar to MSCs. HLSCs also suppress natural killer degranulation, protecting them from allogeneic lysis (Bruno et al., 2016).

In addition to all the preclinical *in vivo* studies, the first human Phase I clinical trial of HLSC in infants with inherited neonatal-onset hyperammonemia demonstrated clinical safety (Spada et al., 2020). In these patients, no immunosuppressive regimen was planned, and no local or systemic adverse events were reported.

We have demonstrated the differentiation capacity of HLSCs towards the hepatic lineage using two approaches: a bioartificial liver (BAL) device (Fonsato et al., 2010) and rat liver acellular scaffolds (Navarro-Tableros et al., 2015). In both approaches, differentiated HLSCs increased the expression of some CYP450 isoforms and secreted urea into the supernatant. Additionally, we have developed a 3D rotary *in vitro* assay to generate functional hepatocyte-like cells from HLSCs (Herrera et al., 2013).

**FIGURE 1:**
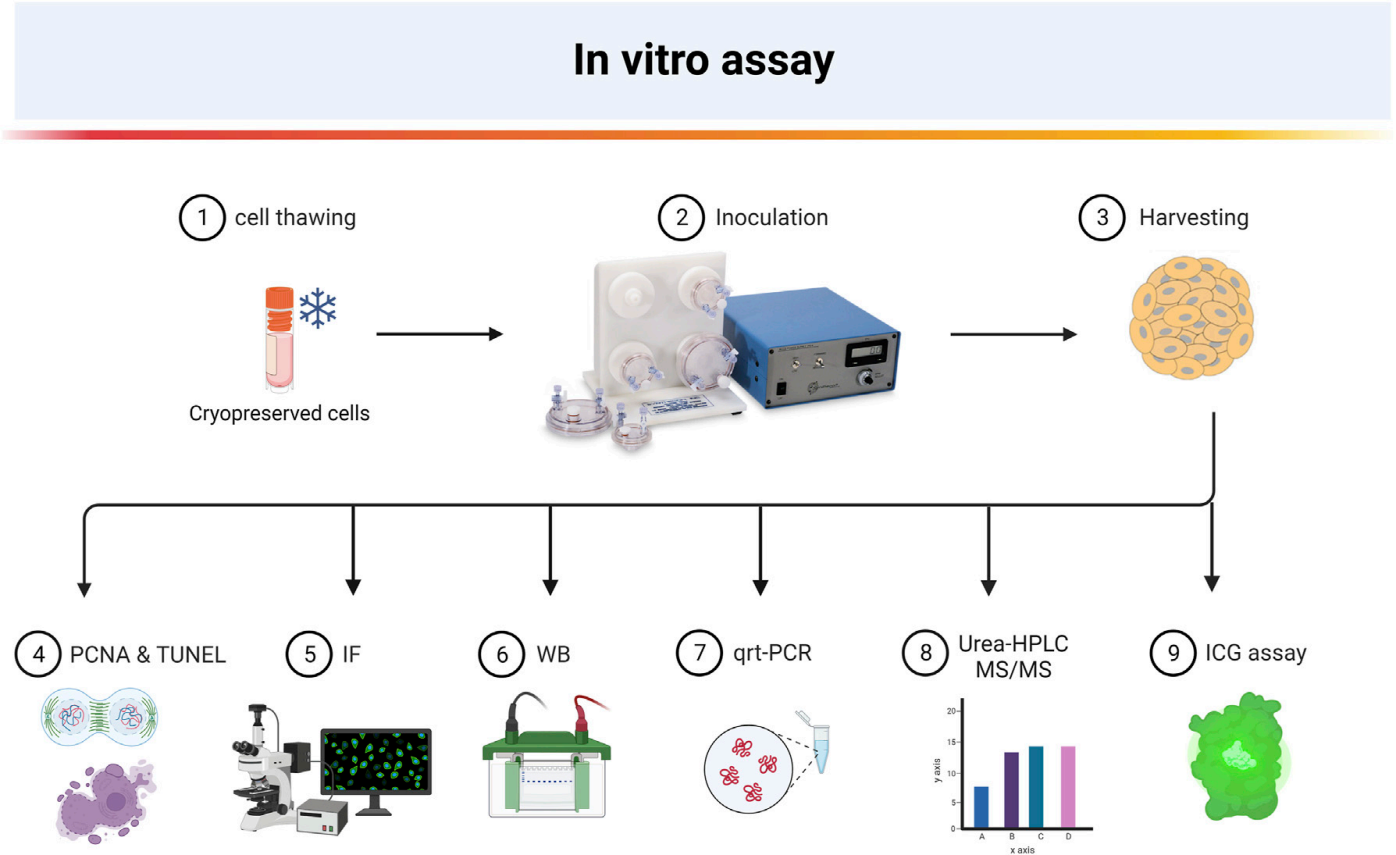
Overview of experimental steps for 3D hepatocyte-like differentiation of HLSC (Created with BioRender.com).

In this study, we better characterized the process of hepatic differentiation in the 3D rotary *in vitro* assay over 10 days of culture. We followed the expression of mature hepatic markers, such as CYP450 cytochromes, urea cycle enzyme, and coagulation factors, as well as pluripotent stem cell markers (Figure 1). Overall, the results obtained could be used for future validation to qualify HLSC as a Good Manufacturing Practice (GMP) drug product.

## 2 Materials and methods

### 2.1 Cell culture

Human liver stem cells were expanded from a GMP-produced master cell bank (MCB-ML-011-01, provided by Unicyte AG, Switzerland). Briefly, HLSC MCB was generated starting from 10 to 15 mm liver fragments obtained from liver donor, according to the standard criteria of Centro Nazionale Trapianti (Spada et al., 2020). Liver tissue was digested with GMP- grade collagenase (0.6 mg/mL) and neutral protease (0.73 mg/mL) (Nordmark Pharma GmbH, Germany) for 20 min at 37°C. The liver cell suspension obtained was washed once (400 g, 10 min) and cultured (4 × 10^4^ cell/cm^2^) in minimal essential medium (α-MEM) (Lonza, Basel, Switzerland) supplemented with 10% fetal calf serum (FCS, Euroclone, Milan, Italy), 4 ng/mL of human recombinant epidermal growth factor (hrEGF, Miltenyi Biotec, Bergisch Gladbach, Germany), 4 ng/mL of human recombinant fibroblast growth factor basic (hrFGF2, Miltenyi Biotec), 2 mM of L-glutamine (Lonza), and 100 U/ml of penicillin/streptomycin (Sigma-Aldrich, St. Louis, MO, United States) in a humidified 5% CO_2_ incubator at 37°C. The HLSCs obtained after 2 weeks of culture were seeded at a density of 3 × 10^3^ cells/cm^2^ in T75 flasks in the same culture medium. Once the cells reached 80% confluency, they were further expanded for 3 passages and then frozen in 5 × 10^6^ cell/vials as MCB stock. MCB-ML-011-01 (passage 4) were subculture in an R&D laboratory for two additional passages until reaching passage 6 (Working cell bank; WCB, p6). Three WCB were generated and subsequently employed for all subsequent experiments. All reagents utilized during the R&D expansion phase met clinical-grade standards and were manufactured in accordance with AIFA regulations.

HaCaT cells were a kind gift from Prof. Calautti (MBC, Turin). Cells were subculture in Dulbecco Modified Eagle Medium (DMEM) high glucose (Thermo Fisher Scientific), supplemented with 2 mM L-glutamine and 10% FCS (Thermo Fisher Scientific). Cells were harvested using 1:1 mixture of 0.05% EDTA and 0.1% trypsin in 1X PBS without Ca_2_+ and Mg_2_+ to maintain physiological osmolality. HepG2 cells (purchased from ATCC, Virginia, United States) were cultured in DMEM supplemented with 10% FCS. Human hepatocytes (Hep) were purchased from Lonza and cultured according to the manufacturer’s protocol.

The H9 cell line was provided by Professor Oliviero from the University of Turin. To prepare the cell culture, we coated 8-chamber slides with Corning Matrigel Matrix-GFR (Corning Incorporated, Somerville, Massachusetts, United States) at a density of 40,000 cells per well. The H9 cell line was cultured in TeSR™-E8™ basal medium supplemented according to the manufacturer’s protocol (STEMCELL Technologies, Canada, Vancouver).

### 2.2 Fluorescence-activated cell sorter analysis

HLSC at passages 6 was characterized by FACS analysis using BD FACSCelesta™ Cell Analyzer (BD Biosciences, Frankin Lakes, NJ, United States). The following antibodies were used: anti CD44, CD105, CD73, CD90, CD29, CD14, CD34, CD45, and human Albumin. FITC, PE or APC conjugated antibodies were used where applicable (Supplementary Table S2). Specific FITC-, PE-, APC-isotype (all from Miltenyi), and human FITC-isotype (for albumin staining-LSBio) were used as negative control. Intracellular staining for Albumin detection was performed using BD Cytofix/Cytoperm™ (BD Pharmingen) according to the manufacturer’s protocol. Data were analysed by BD FACSDiva™ Software (BD Biosciences).

### 2.3 *In vitro* hepatic 3D cell differentiation in rotary cell culture system

HLSC were differentiated into hepatocyte-like cells using a modified version of a previously described protocol (Herrera et al., 2013). Briefly, 5 × 10^6^ cells were resuspended in 10 mL of differentiation media composed of: 60/40 (v/v) mixture of low glucose DMEM (without phenol red, Euroclone) and MCDB-201 (without phenol red, Cell technologies, Lugano, Switzerland), supplemented with 2% FCS (Thermo Fisher Scientific), 0.026 g/mL ascorbic acid 3-phosphate, linoleic acid-bovine serum albumin (LA-BSA), insulin-transferrin-selenium (ITS) (all from Sigma), 10 ng/mL rhFGF4 and, 10 ng/mL rhHGF (both from Miltenyi) (Herrera et al., 2013). Cells were inoculated in the rotary vessels (Synthecon, Houston, TX, United States) and rotated at 8 rpm/min in a clockwise direction. After 1, 4, 7, and 10 days of culture, the supernatant, including the cell aggregates, was centrifuged at 300 *g* for 5 min. The supernatants were collected, aliquoted and stored at −20°C, while the HLSC aggregates were washed once with PBS and used for the different experiments.

### 2.4 Terminal dUTP nick end-labeling assay

Apoptosis was evaluated using the terminal dUTP nick end-labeling (TUNEL) assay (Merck Millipore, Darmstadt, Germany) in HLSCs and differentiated HLSC aggregates. Briefly, 10 µm sections of OCT-embedded aggregates were sliced. Next, the sections were fixed with precooled ethanol/acetic acid (2:1) for 5 min at room temperature. HLSCs were cultured in chamber slides (Nalgene Nunc International, Rochester, NY) and fixed in 4% paraformaldehyde before used.

The aggregates and HLSCs were then treated with terminal deoxynucleotidyl transferase enzyme and incubated in a humidified chamber for 1 h at 37 °C. Next, the sections were treated with fluorescein isothiocyanate-conjugated anti-digoxigenin for 30 min at room temperature, according to the manufacturer’s instructions. After washing, the samples were mounted in SlowFade Gold antifade reagent (Thermo Fisher Scientific, Eugene, OR, United States), and the cells were analyzed by immunofluorescence microscopy using Leica SP8 Confocal Microscope (Wetzlar, Germany) with Leica navigator software. The TUNEL-positive and total nuclei were counted using ImageJ software (https://imagej.nih.gov/).

### 2.5 Periodic acid schiff staining

Paraffin sections of HLSC aggregates were deparaffinized, hydrated in water, and rinsed with water. The sections were then incubated with 0.5% periodic acid solution for 5 min, stained with Schiff’ reagent for 15 min, and counterstaining with hematoxylin solution for 2 min. All steps were performed at room temperature.

### 2.6 Immunofluorescence staining

Immunofluorescence staining was performed as described previously (Herrera et al., 2006). Briefly, HLSC cultured in chamber slides (Nalgene Nunc International, Rochester, NY) were fixed in 4% paraformaldehyde, permeabilized with Hepes-Triton X-100 buffer (Sigma-Aldrich), and then stained as follows. HLSC aggregates recovered from RCCS at day 1, 4, 7, and 10 were directly embedded in OCT (Bio-Optica, Milan, Italy) and cut into 10 µm slices. Nonspecific antibody binding in the cells and slices was blocked by incubation in PBS solution +1% bovine Serum Albumin (BSA; Sigma-Aldrich) for 1 h at room temperature.

Aggregates, sections, or cells were stained with the following primary and secondary antibodies (Supplementary Table S2): Primary antibodies: anti-OCT3/4, anti-Nanog, anti-Sox2, anti-KLF4, anti-HNF4a, anti-human albumin, anti-OATP1B1, anti-cytokeratin 18, anti-cytokeratin 8, anti-aFP, anti-CYP1A1, anti-CYP3A4, anti-CYP7A1, anti-PCNA, anti-OTC, anti-ASS1, anti-CPS1, anti-ARG1, anti-ASL, anti-FVIII, anti-FIX and anti-FXI (Supplementary Table S2). Secondary antibodies: Alexa Fluor 488 goat anti-mouse IgG, anti-rat, and donkey anti-goat; and Alexa Fluor 594 chicken anti-mouse IgG (All from Thermo Fisher Scientific, Waltham, MA, USA). Omission of the primary antibodies or substitution with nonimmune rabbit, rat, or mouse IgG served as control (Supplementary Table S2; Supplementary Figure S8). Confocal microscopy analysis was performed using Leica SP8 Confocal Microscope (Wetzlar, Germany). SlowFade Gold antifade reagent (Thermo Fisher Scientific, Eugene, OR, United States) was used for nuclear staining in combination with labeling and detection flour mount solution. HepG2 and human hepatocyte were used as positive control for the urea cycle enzymes expression (Supplementary Figures S7A, C).

### 2.7 Western blot

The cell aggregates were collected from RCCS and centrifuged at 400 *g* for 5 min at room temperature. The supernatants were aliquoted, and the cell aggregates were washed once with PBS 1×. After washing, the PBS was removed, and the aggregates were disrupted in liquid nitrogen and resuspended in 70 µL of RIPA buffer (RIPA + PMSF + protease inhibitors, Sigma-Aldrich). The samples (HLSCs and HLSC aggregates) were kept on ice for 15 min to induce cell lysis and then centrifuged at 12,000 g for 15 min at 4°C. For protein quantification, the PierceTM BCA Protein Assay Kit (Thermo Fisher Scientific) was used according to the manufacturer’s protocol, and the absorbance was read at 562 nm on an iMark Microplate reader (Bio-Rad, Hercules, CA, United States). Extracted proteins were then analyzed by SDS-PAGE, and the membranes were incubated with specific primary antibodies against urea cycle enzymes (Supplementary Table S2). GAPDH and vinculin were used to normalize the data. Results were presented as density units and analyzed with ImagLab software (Bio-Rad). HepG2 were used as positive control for urea cycle enzymes (Supplementary Figure S7B). For the analysis of FVIII release into the supernatant, the samples were first processed with 50 kDa filter spin columns (Millipore, Burlington, MA, United States) and then the flowthrough was concentrated hundred-fold using 10kD filter spin columns (Abcam) according to the manufacturer’s instructions. Total proteins quantification is shown in Supplementary Table S6.

### 2.8 Gene expression analysis by real time PCR

RNA was extracted from HLSC at passages 6/7 and RCCS aggregates at day 1 and day 4 using the miRNeasy Micro Kit (Qiagen, Hilden, Germany) according to the manufacturer’s protocol. The RNA concentration was measured using a Nanodrop ND-2000 (Thermo Fisher Scientific), and eluted RNA was stored at −80°C until further use. The quantification values are shown in Supplementary Table S4.

To screen for liver-related genes, a custom RT^2^ Profiler PCR Array designed on the Qiagen GeneGlobe website (Qiagen) was used. For the analyses, 1 µg of total RNA was retro-transcribed using the RT^2^ First Strand Kit (Qiagen) according to the manufacturer’s protocol. The cDNA, along with RT^2^ SYBR Green ROX™ qPCR Mastermix, was applied to the custom PCR array cards and run on the StepOnePlus real-time PCR instrument (Applied Biosystem, Foster City, CA) according to the manufacturer’s instructions. Five endogenous controls (ACTB, HPRT1, GUSB, GAPDH, B2M) were selected, and retrotranscription, genomic contamination, and reproducibility controls were added. Data were analyzed using the online GeneGlobe software, and results were provided as means of fold Change (2^−ΔΔCt^) or with Expression Suite software (Applied Biosystem). ΔΔCt is the normalized gene expression (2^−ΔCt^) in the test sample divided by the normalized gene expression (2^−ΔCt^) in the control sample. Biological replicates of each condition (*n* = 5) were run in duplicate. The complete list of analyzed genes is provided in Supplementary Table S3.

For specific gene expression analysis, quantitative real-time PCR was performed. Briefly, first-strand cDNA was synthesized from 200 ng of total RNA using the High-Capacity cDNA Reverse Transcription Kit (Applied Biosystems). qRT-PCR was performed using the StepOnePlus machine (Applied Biosystems) in a 20 μL reaction mixture containing 5 ng or 10 ng of cDNA template (according to tested gene expression analysis), the sequence-specific oligonucleotide primers (purchased from MWG Biotech, Eurofins Scientific, Brussels, Belgium), and the Power SYBR Green PCR Master Mix (Applied Biosystems). TBP was used as endogenous control. Fold change expressions with respect to control were calculated for all samples using the ΔΔCt method. The primers used for qRT-PCR are reported in Supplementary Table S1.

### 2.9 Urea quantification by HPLC-MS/MS analysis and quantichrom urea assay kit

Urea production was measured by liquid chromatography hyphenated with tandem mass spectrometry (LC-MS/MS). Urea chromatographic separations were performed on a Nexera HPLC (Shimadzu, Kyoto, Japan) coupled to a Qtrap mass spectrometer (Sciex 5500, Framingham, MA, United States) equipped with an ESI Turbo Ion Spray source. Supernatant samples from RCCS experiments were analyzed using a hydrophilic interaction liquid chromatography (HILIC) column (Phenomenex Luna HILIC 150 × 2.1 mm, 3 μm particle size, Phenomenex, Torrance, CA, United States) at a flow rate of 200 μL/min. A gradient mobile phase composition was used: 95/5 to 20/80 solvent A/solvent B. Solvent A was acetonitrile and solvent B was 5 mM aqueous ammonium acetate with 2% of acetonitrile. The injection volume was 20 μL. The tuning parameters used for the ESI source were: source voltage 4.5 kV (positive ion mode), source temperature 300°C, and curtain gas nitrogen. Analyses were performed using selected reaction monitoring MS/MS acquisition, with a precursor ion 61 m*/z*, a product ion 44 m*/z*, and a collision energy 20 V. The lower limit of quantification (LLOQ) was 50 ng/mL (supernatant samples were tenfold diluted with acetonitrile before injection). Data were expressed as parts per million (ppm, µg/mL). An external urea standard curve (Sigma-Aldrich) was used to quantify the concentration of the samples.

To further quantify urea levels, we employed the QuantiChrom™ Urea Assay Kit-DIUR 100 (BioAssay Systems, CA, United States). This kit utilizes an improved Jung method that directly measures urea in supernatant without the need of prior treatment. The method employs a chromogenic reagent that forms a specific-colored complex with urea. The absorbance of the resulting color, measured at 520 nm, was directly proportional to the urea concentration in the sample.

### 2.10 Hepatocyte functional assay: indocyanine green uptake

Indocyanine green (ICG) (ICG, USP, Frederick, MD, United States) was dissolved in DMSO at 33 mg/mL and added to alpha MEM (without Phenol red, Lonza, Basel) to a final concentration of 0.5 mg/mL. The cells or aggregates were incubated with ICG at 37°C for 60 min. After incubation, the medium with ICG was blocked with FCS and discarded. The cells or aggregates were washed three times with PBS and examined under the microscope to check for cellular uptake of ICG. Representative images were taken using the Moticam BTX8 Camera (Motic, Xiamen, China). The quantification of ICG uptake was performed by lysing the aggregates or cells with 400 μL of RIPA buffer and measuring the absorbance at 750 nm with a plate reader (iMark Microplate reader, Bio-Rad). The ICG concentration was calculated by plotting a concentration vs*.* absorbance standard curve of ICG. Measurements were performed in duplicates. HaCaT and non-differentiated HLSC were used as negative control, while hepatocytes (Lonza) were used as a positive control.

### 2.11 Statistical analysis

Raw data were analyzed using GraphPad Prism 6.0 software (GraphPad Software, San Diego, CA, United States). Results are expressed as the mean ± standard deviation of 3-5 independent biological replicates. Statistical analyses were performed using one-way analysis of variance (ANOVA) followed by Newman–Keuls comparisons test or Student’s t-test, as appropriate. A *p*-value of <0.05 was considered statistically significant.

## 3 Results

### 3.1 HLSC characterization cultured in 2D condition

**FIGURE 2:**
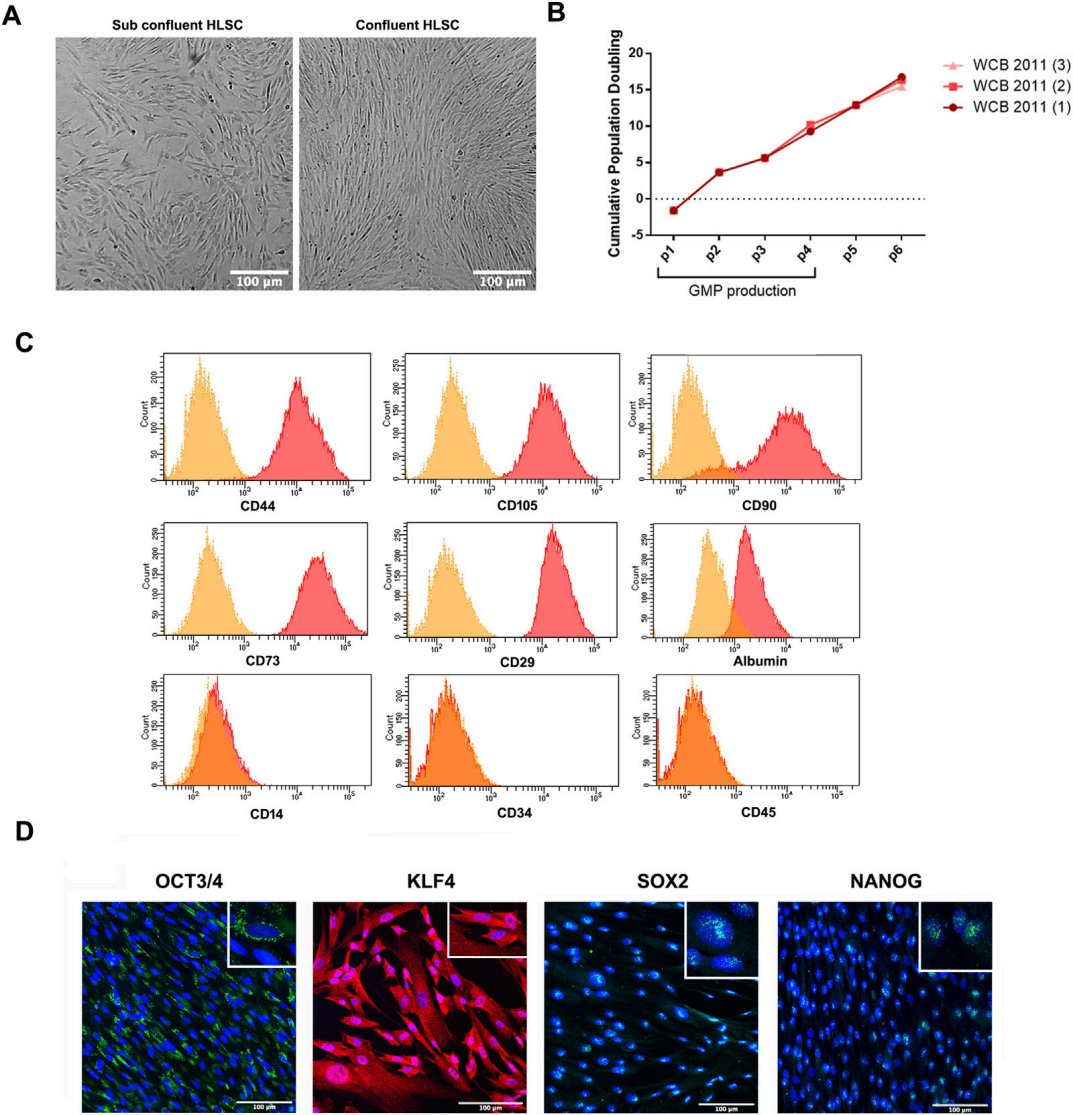
HLSC characterization. **(A)** Representative micrograph showing HLSC morphology. **(B)** Cumulative population doubling (CPD) of three different cell batches of HLSCs from passage 0 to 6. **(C)** Representative FACS analysis for the positive surface markers CD44, CD105, CD73, CD90, CD29 and albumin and the negative markers CD34, CD45, and CD14. **(D)** Representative immunofluorescence micrograph of stem cell markers Nanog, SOX2, KLF4, OCT3/4 in undifferentiated HLSCs. Scale bar, 100µm; magnification × 40, insert pictures. Nuclei are stained in blue with DAPI.

HLSCs were isolated from a liver biopsy under GMP conditions (Spada et al., 2020) and expanded and frozen in a working cell bank (WCB) at passage 6. During expansion, HLSC-WCB maintained a spindle-shaped morphology without any signs of overgrowing or cell suffering, reaching over 80% confluency (Figure 2A). The proliferative potential and cell bank reproducibility were evaluated by measuring the cumulative population doubling (CPD) of the cells after expansion in 2D. As shown in Figure 2B, no statistically significant differences in CPD were observed up to passage 6 (Figure 2B).

To confirm the identity of the HLSCs, a panel of surface markers were analyzed by fluorescence-activated cell sorter (FACS) and immunofluorescence analysis. The analysis showed that HLSCs expressed high levels (≥90%) of CD29, CD73, CD90, CD105, and CD44, while lacking expression of the immune cell marker CD14, the hematopoietic cell marker CD45, and the hematopoietic stem cell marker CD34. Additionally, albumin expression was confirmed (Herrera et al., 2013) (Figure 2C).

Consistent with previous immunofluorescence analysis, HLSC expressed OCT3/4, SOX2 and Nanog (Herrera et al., 2013) and we further observed KLF4 expression in these cells (Figure 2D). Nanog and SOX2 were localized in the nucleus of HLSCs, KLF4 was found in the cytoplasm, and OCT3/4 was localized in the perinuclear region (Figure 2D). H9 embryonic cell lines was used as positive control for stem cell markers (Supplementary Figure S1).

### 3.2 Characterization of HLSC aggregates in rotary cell culture system

**FIGURE 3:**
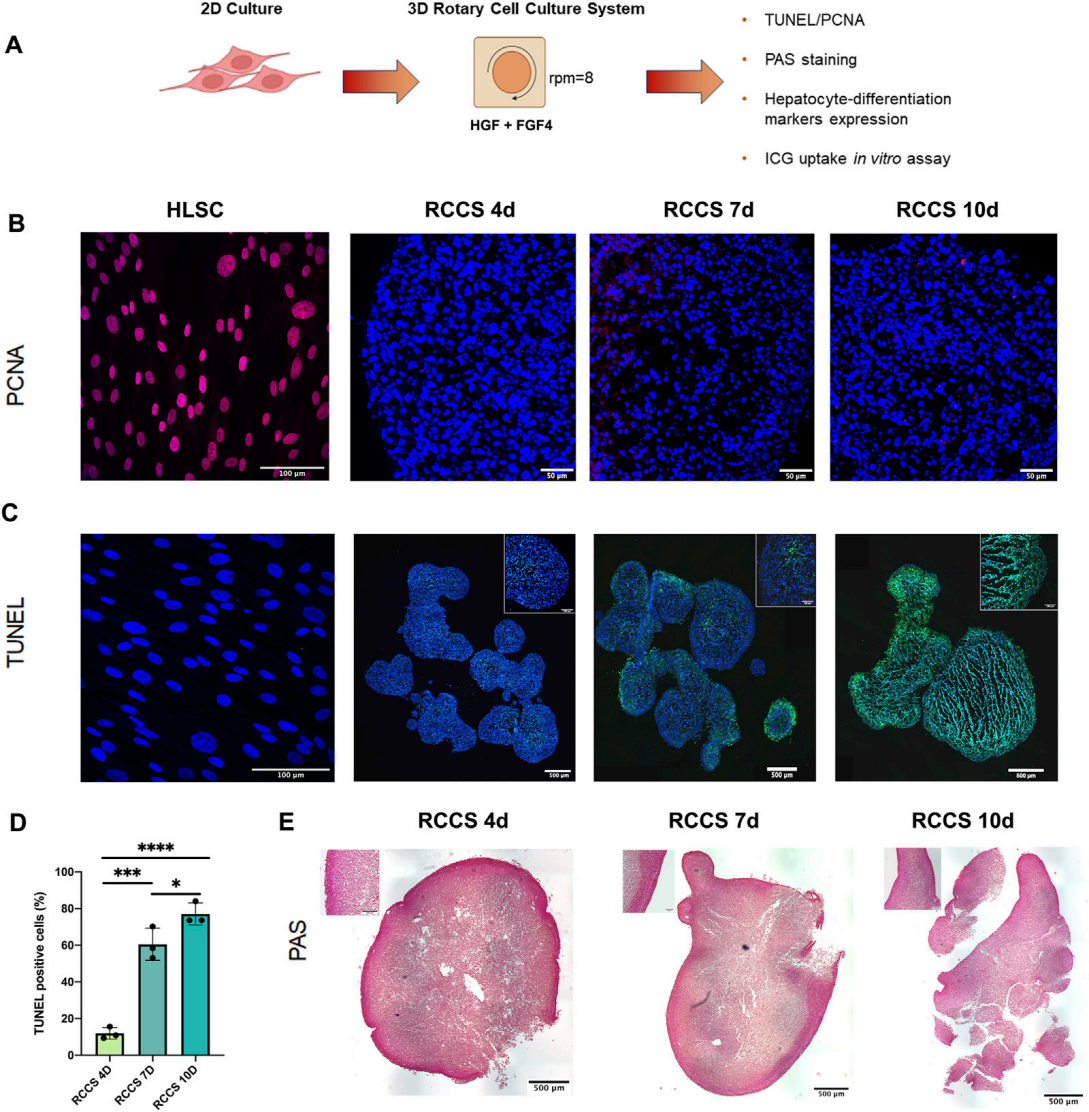
*In vitro* differentiation of HLSCs into hepatocyte-like cells in RCCS. **(A)** Experimental layout. **(B)** Representative PCNA staining of undifferentiated HLSC and differentiation HLSCs in RCCS after 4-, 7- and 10-days. Scale bar, 50, 100 µm. × 40 magnification. HLSCs were used as a positive control. Nuclei (blue) were stained with DAPI. **(C)** Apoptotic cell death was detected by TUNEL (green), and the nucleus (blue) was stained with DAPI. Scale bar, 500 μm; insert pictures, 100 µm. × 20 magnification. HLSCs were used as an internal control. **(D)** Quantification of TUNEL-positive cells in all the experimental groups. Values represent the mean ± SD from three independent experiments (black dots represent the experimental replicates). ***p < 0.0001 RCCS 4d vs*.* RCCS 7d, *p < 0.05 RCCS 7d vs. RCCS 10d, ****p < 0.001 RCCS 4d vs*.* RCCS 10d. **(E)** PAS staining of RCCS aggregates analyzed at day 4, 7, and 10 post-differentiation. Scale bar, 500µm, insert pictures, 100 µm, ×20 magnification.

Figure 3A shows a schematic overview of the experiment. HLSCs were maintained in differentiation conditions until the end of the assay. Cell aggregates and HLSCs were analyzed for proliferating cell nuclear antigen (PCNA) and TUNEL. In addition, Periodic acid–Schiff (PAS) staining was performed after 4, 7 and 10 days of HLSC culture in RCCS (Figure 3). PCNA staining revealed no proliferation of HLSCs during differentiation compared to undifferentiated HLSCs (Figure 3B). Apoptosis measured by TUNEL staining, was evident in HLSC aggregates, with a gradual increase from 11.9% ± 3.2% on day 4%–77.1% ± 6.1% on day 10 (Figures 3C,D). In contrast, on undifferentiated HLSC, no apoptotic cells were observed (Figure 3C). This observation was further corroborated by a significant reduction in total RNA content from HLSCs after 7 days in RCCS, confirming cell loss during this period (Supplementary Table S4).

PAS staining, a marker of glycogen synthesis and hepatocyte function, showed PAS-positive areas on the surface of the aggregates, suggesting the presence of glycogen-producing cells on the outer part of the structure (Figure 3E). Based on the TUNEL and PAS staining results, we decided to focus up to day 4 as the optimal time point for further analysis. For comparison, some experiments were also conducted on day 7.

### 3.3 Differential gene expression analysis by PCR array

**FIGURE 4:**
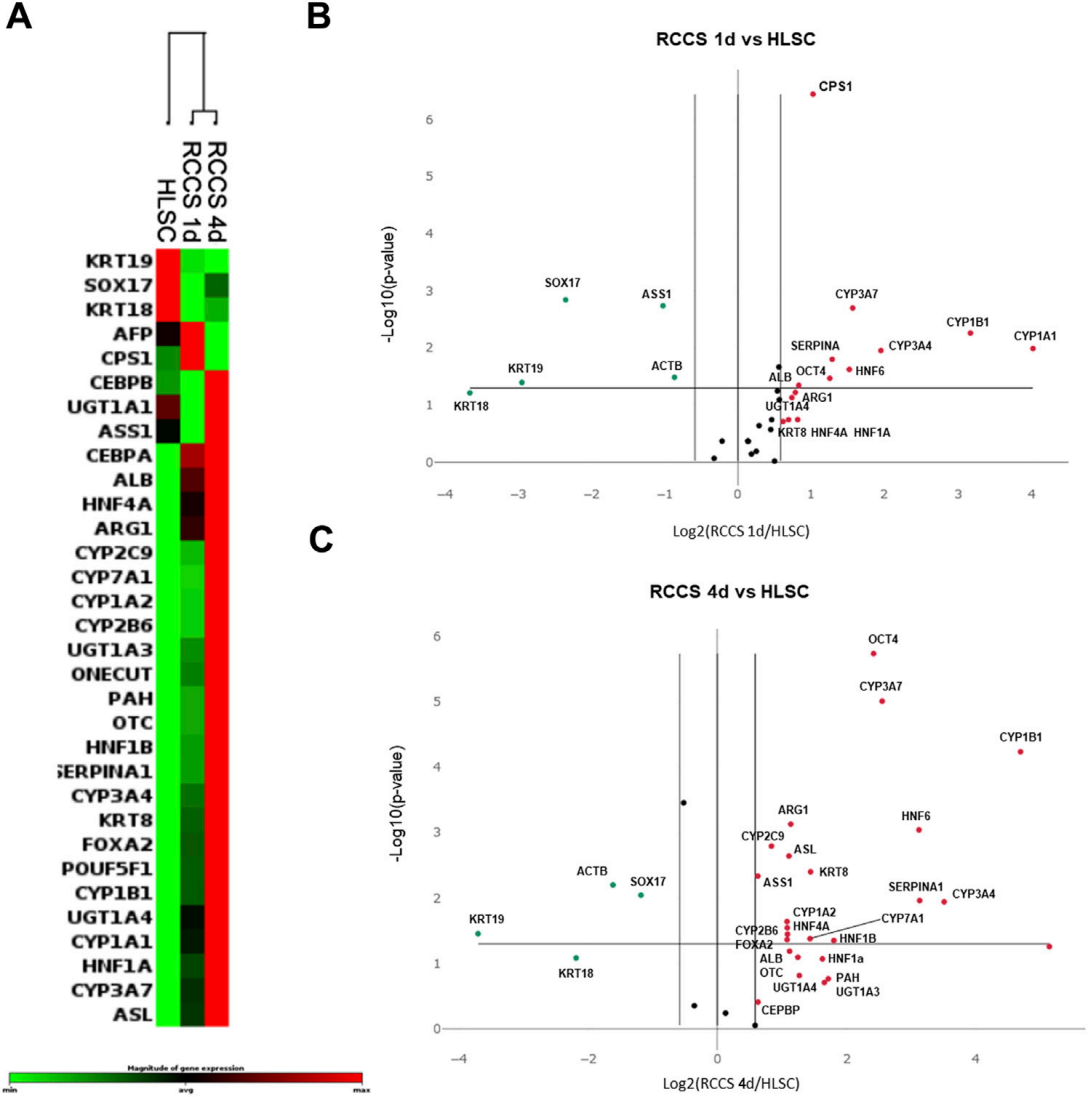
PCR array Gene expression analysis. **(A)** Heatmap of gene expression of the three biological groups analyzed (*n* = 5: number of independent experiments x group); RCCS 1d, and RCCS 4d cluster together, and undifferentiated HLSCs are separated from RCCS samples. **(B)** Volcano plot comparing gene expression of HLSCs vs. RCCS day 1 and **(C)** vs. RCCS day 4. Red dots represent upregulated genes and green dots represent downregulated genes. All dots above the *p*-value line are statistically significant with respect to control.

To evaluate the maturation state of HLSCs, we compared the gene expression profiles of undifferentiated and RCCS-differentiated HLSCs using PCR array analysis (Figure 4; Supplementary Table S5; Supplementary Figure S2). We used human hepatocytes as positive control of fully differentiated cells (Supplementary Table S5).

Our findings revealed distinct gene expression patterns between undifferentiated and differentiated HLSCs. Clustering analysis demonstrated significant changes in the expression of hepatic genes across all groups analyzed (Figure 4; Supplementary Table S5; Supplementary Figure S2).

Upon exposure to RCCS, 13 out of 32 genes exhibited significant modulation (9 upregulated and 4 downregulated) in HLSCs after day 1, while 19 out of 32 genes showed significant modulation (15 upregulated and 4 downregulated) after 4 days (Figures 4B,C; Supplementary Table S5; Supplementary Figure S2). The majority of these differentially expressed genes encode hepatic signaling molecules involved in liver development and are typically expressed in mature hepatocytes (Figure 4; Supplementary Table S5).

The list of mature hepatocyte upregulated genes in RCCS differentiated HLSCs, included KRT8, UGT1A3, UGT1A4, POUF5F1, FOXA2, CEBPB, HNF1A, HNF4A, HNF6, ALB, PAH, CYP1A1, CYP1A2, CYP1B1, CYP2B6, CYP3A4, CYP3A7, CYP7A1, SERPINA1, CPS1, OTC, ARG1, and ASL (Figure 4; Supplementary Table S5; Supplementary Figure S2).

Subsequently, we examined the expression of both proteins and genes associated with hepatic differentiation in RCCS-treated HLSCs compared to undifferentiated HLSCs.

### 3.4 Immunofluorescence and rt-PCR analysis of hepatocyte-like differentiated HLSCs

**FIGURE 5:**
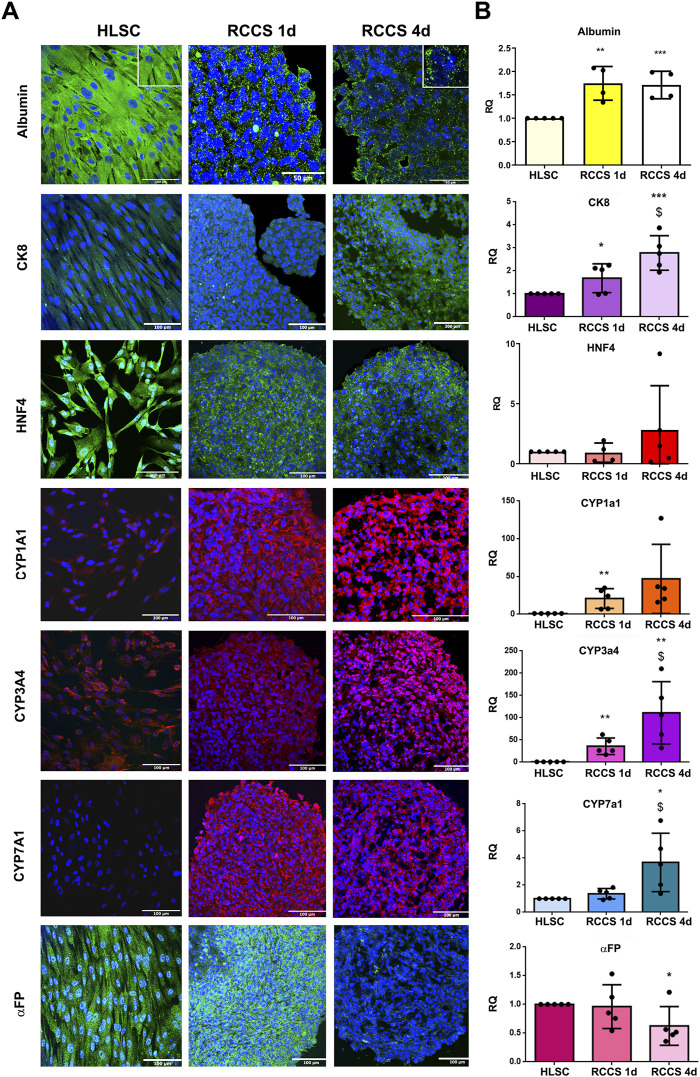
Hepatic markers. **(A)** Representative immunofluorescence micrographs of Albumin, CK8, HNF4, cytochrome P450 isoform CYP1a1, CYP3a4, CYP7a1 and αFP, in HLSCs and HLSC aggregates after differentiation in RCCS. Scale bar, 50 and 100 µm; magnification ×40, insert pictures (albumin staining). Nuclei are stained in blue with DAPI. **(B)** Real-time PCR array data analysis of hepatic markers of undifferentiated and differentiated HLSCs. Results are expressed as Relative Quantification (RQ), data were normalized using TBP and undifferentiated HLSCs were used as experimental control. The results are presented as mean values of five independent experiments ±SD *p < 0.05, **p < 0.01, ***p < 0.001 RCCS 1d and 4d vs*.* HLSC, ^$^ *p* < 0.05 RCCS 4d vs*.* RCCS 1d.

To assess the efficiency of our hepatic differentiation system, we characterized HLSC aggregates by evaluating the expression of specific liver markers (Figure 5). As previously demonstrated (Herrera et al., 2013), albumin was highly expressed in undifferentiated HLSCs (Figure 5A). During differentiation, albumin expression remained stable in RCCSs (Figure 5A), exhibiting a transition from cytoplasmic localization to granular localization after 1 day. On the other hand, albumin mRNA expression exhibited a twofold increase during differentiation in RCCS (Figure 5B).

HNF4, a transcription factor crucial for liver development, was well expressed in undifferentiated HLSCs and maintained throughout differentiation, as shown in Figure 5A. This was further confirmed by no differences in mRNA expression (Figure 5B). CK8, a marker of mature hepatocytes, was expressed at low levels in undifferentiated HLSCs and increased significantly during differentiation in RCCS at both protein and gene levels (Figures 5A,B).

Cytochrome P450 (CYP) enzymes are essential for drug metabolism and a hallmark of mature hepatocytes. Specific CYP isoforms, including CYP1a1, CYP3a4, and CYP7a1, were expressed at low levels in HLSCs and increased during differentiation (Figures 5A,B). The PCR array further revealed that the gene expression of CYP1b1 and CYP3a7 increased after 1 day of differentiation and remained elevated after 4 days, while CYP1a2 and CYP2b6 expression was significantly upregulated at day four (Supplementary Table S5).

αFP, an immature hepatoblast marker (Elmaouhoub et al., 2007), was significantly reduced in differentiated HLSCs compared to undifferentiated HLSCs at both protein and gene levels (Figures 5A,B).

Stem cell markers Nanog, SOX2, and OCT3/4 exhibited dynamic expression patterns during differentiation. Their gene expression peaked at day four in RCCS 3D aggregates (Supplementary Figure S3A) and then declined after 7 days. Additionally, the protein expression pattern of Nanog and OCT3/4 changed from nuclear in undifferentiated HLSCs to cytoplasmic in differentiated HLSCs. In contrast, KLF4 expression decreased over time in RCCS (Supplementary Figure S3A), correlating with the gene expression level (Supplementary Figure S3B).

These results demonstrate that RCCS effectively induces hepatic differentiation in HLSCs, leading to the expression of key liver markers and functional enzymes.

Human hepatocytes were employed as positive controls for fully differentiated cells, as evidenced by their expression of albumin, CYP1A1, CYP3A4, CYP7A1, CK8, and FVIII (Supplementary Figure S4). HNF4 and αFP, on the other hand, exhibited lower expression levels in human hepatocytes (Supplementary Figure S4).

### 3.5 Expression of coagulation factors

**FIGURE 6:**
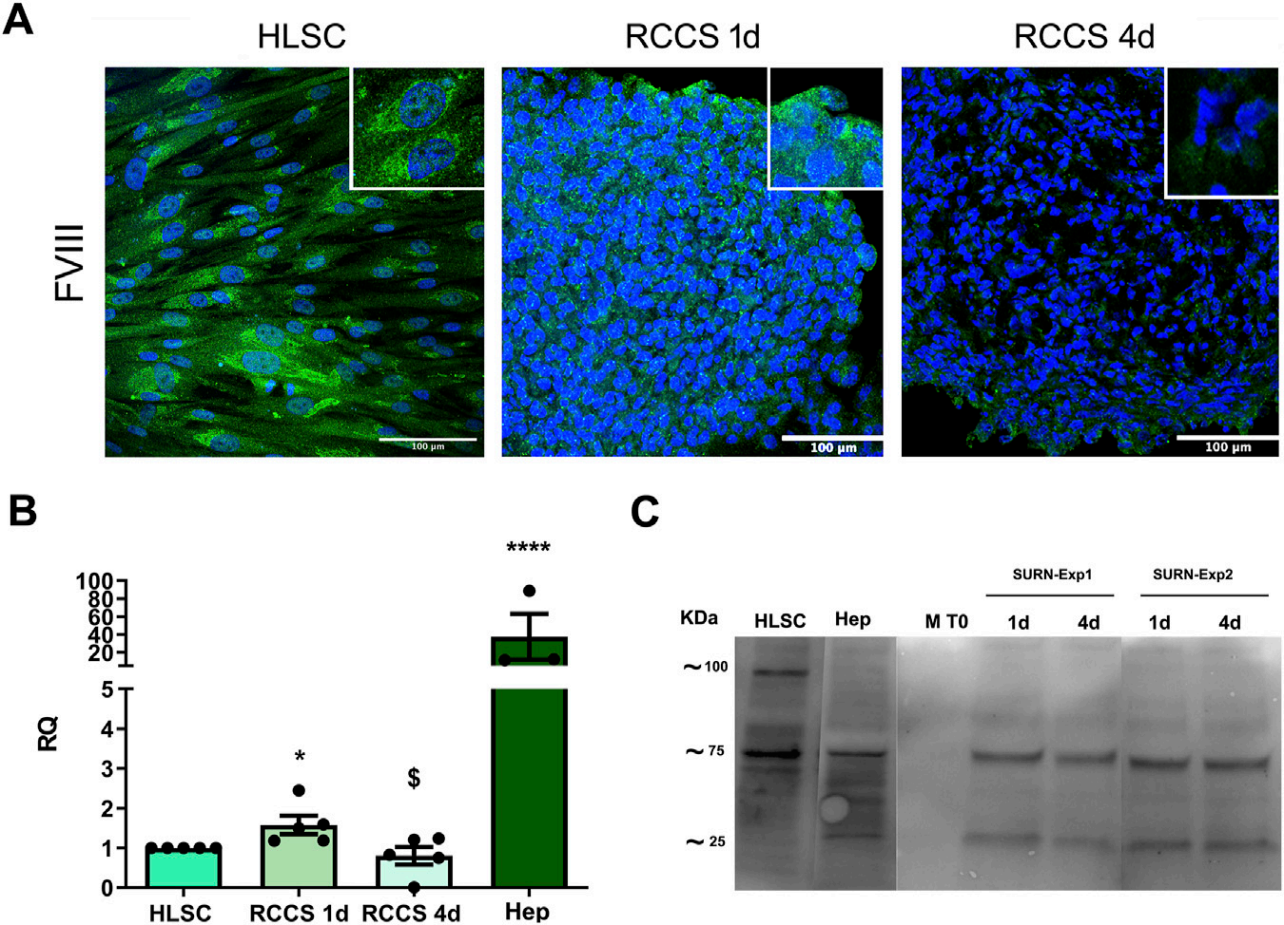
Expression of Coagulation Factors. **(A)** Representative immunofluorescence micrographs of coagulation factor FVIII in HLSCs and differentiated HLSC in RCCS after 1 and 4 days. Scale bar, 100 µm. Magnification × 40, insert pictures. Nuclei are stained in blue with DAPI. **(B)** Real-time PCR analysis of FVIII in HLSC, HLSC differentiated after 1 and 4 days, and human hepatocyte (Hep). Results were normalized using TBP and undifferentiated HLSCs were used as experimental control. Real time data are expressed as Relative Quantification (RQ). The results are presented as mean values of five independent experiments ±SD. *p < 0.05 RCCS 4d vs. HLSC; ^$^ *p* < 0.05 RCCS 4d vs*.* RCCS 1d, ****p < 0.0001 Hep vs*.* HLSC. **(C)** Representative Western Blot of FVIII in HLSCs, human hepatocyte (Hep) and HLSC supernatants after differentiation. M T0: differentiation media at t0 (without HLSCs), 1d and 4d: are supernatants collected after 1 and 4 days of HLSC differentiation in RCCS from two different experiments (SURN-Exp1 and SURN-Exp2).

We evaluated the expression of coagulation factors FVIII, FIX, and FXI in HLSCs and differentiated HLSCs using indirect immunofluorescence (Figure 6; Supplementary Figure S5). HLSCs exhibited strong positive staining for FVIII (Figure 6A) and FIX (Supplementary Figure S5A), while FXI expression was lower (Supplementary Figure S5A). Notably, FVIII expression decreased significantly in differentiated HLSCs both at the protein and gene levels after 4 days of differentiation (Figures 6A,B). The FVIII mRNA expression in human hepatocytes was approximately ten times higher compared to differentiated HLSCs (Figure 6B).

To investigate whether this reduced expression was associated with FVIII secretion, as observed in hepatocytes, we measured FVIII levels in the culture supernatant. We found that the decline in FVIII expression in the 3D aggregates correlated with an increase in FVIII release into the supernatant (Figure 6C). In contrast to FVIII, the expression of FIX and FXI (Supplementary Figures S5A,B) was significantly higher at both the protein and gene levels in RCCS-treated HLSCs compared to undifferentiated HLSCs (Supplementary Figure S5). These findings suggest that HLSCs can produce coagulation factors, with FVIII secretion increasing during differentiation.

### 3.6 Urea metabolism after differentiation

**FIGURE 7:**
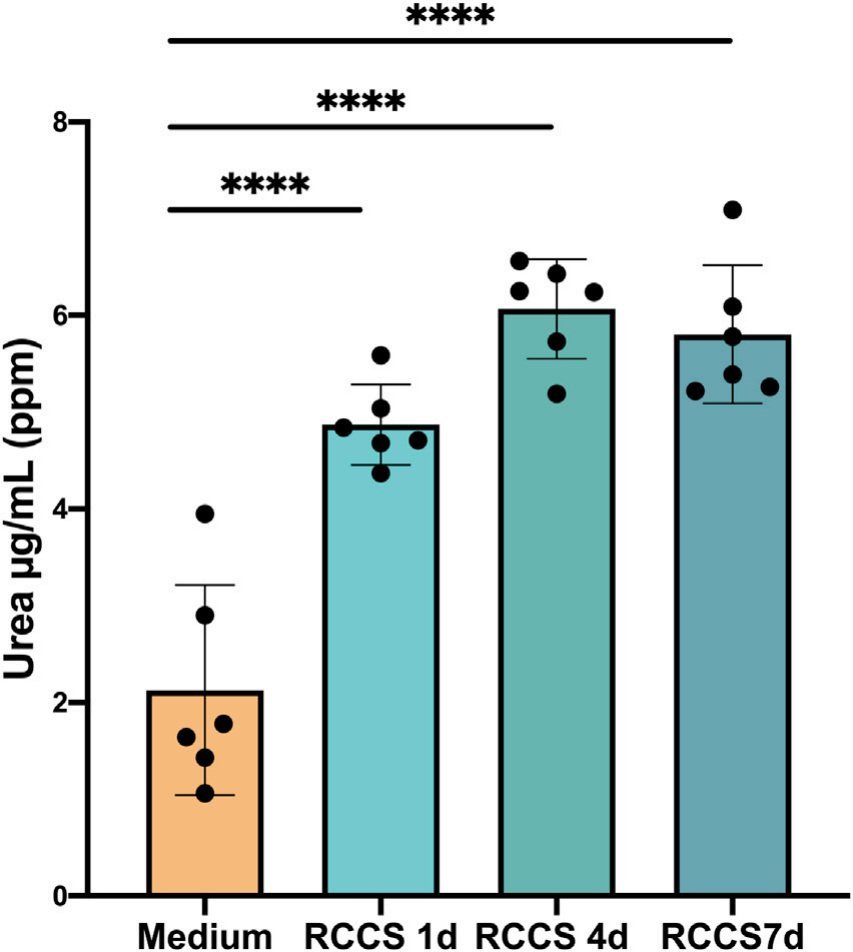
Increase in hepatocyte function of HLSCs under differentiation culture condition. Urea secretion was quantified by HPLC MS/MS analysis. Results were expressed as ppm (part per million) (µg/mL) of urea. The results are presented as mean values of six independent experiments ±SD. ****p < 0.0001 RCCS 1,4,7d vs*.* Medium (without HLSCs).

The urea cycle, the primary mechanism for eliminating waste nitrogen from protein turnover, is predominantly localized to the liver (Barmore and Stone, 2023). This energy-dependent process, initiated in the mitochondria and completed in the cytoplasm, plays a crucial role in maintaining nitrogen balance and preventing ammonia toxicity (Barmore and Stone, 2023). Malfunctioning of the urea cycle leads to the accumulation of toxic ammonia (NH4^+^), resulting in clinical manifestations such as lethargy, slurred speech, cerebral edema, and asterixis (Ah Mew et al., 1993; Soria et al., 2019). To assess the functionality of the urea cycle in differentiated HLSCs, we measured urea secretion in RCCS supernatants by mass spectrometry. Compared to medium alone, urea secretion was significantly higher after day 1 and remained unchanged until day 7 (Figure 7). These findings suggest that differentiated HLSCs are capable of urea synthesis and secretion, with the majority of production occurring early in the differentiation process.

To compare urea secretion in differentiated HLSCs to human hepatocytes, we employed also a colorimetric assay kit (Supplementary Figure S6). The analysis revealed that the amount of urea secreted into the supernatant after 4 days of differentiation was approximately 0.5 mg/dL, significantly lower than the 9.5 mg/dL observed in mature hepatocytes. This finding suggests that HLSCs still undergo limited differentiation after 4 days of induction.

**FIGURE 8:**
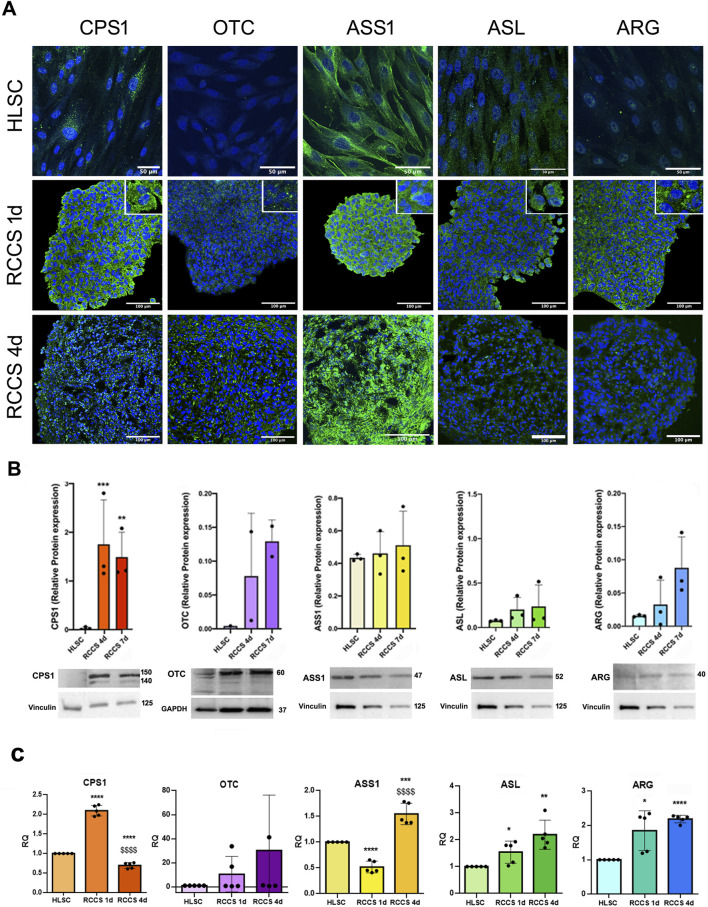
Expression of urea cycle enzymes. **(A)** Representative immunofluorescence micrographs of the urea cycle enzymes CPS1, OTC, ASS1, ASL and ARG in HLSCs and differentiated HLSCs in RCCS after 1 and 4 days. Scale bar, 50 and 100 µm, magnification × 40, insert pictures. Nuclei are stained in blue with DAPI. **(B)** Representative blot and relative amounts of proteins counted in relation to HLSC. The results are presented as mean values of three independent experiments and are normalized using Vinculin and GAPDH as housekeeping proteins ±SD. ***p < 0.001 RCCS 4d vs. HLSC, **p < 0.01 RCCS 7d vs. HLSC. **(C)** Real-time PCR array data of urea cycle enzyme genes in HLSCs and differentiated HLSCs. Results were normalized using TBP and undifferentiated HLSCs were used as experimental control. Data are expressed as Relative Quantification (RQ) of five independent experiments ±SD. *p < 0.05, **p < 0.01, ***p < 0.001,****p < 0.0001 RCCS 1d and RCCS 4d vs*.* HLSC, ^$$$$^ *p* < 0.0001 RCCS 4d vs*.* RCCS 1d.

We further evaluated the expression of urea cycle enzymes in undifferentiated and differentiated HLSCs (Figure 8). Undifferentiated HLSCs exhibited low expression levels of Carbamoyl-Phosphate Synthetase 1 (CPS1), Arginino-succinate lyase (ASL), and Arginase 1 (ARG1), while Arginino-succinate Synthase 1 (ASS1) expression was high, consistent with previous findings (Herrera Sanchez et al., 2017). Notably, Ornithine Transcarbamylase (OTC) was not detected in undifferentiated HLSCs (Figure 8A).

Immunofluorescence and Western blot analyses revealed that the protein levels of all five urea cycle enzymes increased 1 day after differentiation (Figures 8A,B). However, relative protein expression between undifferentiated HLSCs and HLSC aggregates in RCCS remained relatively unchanged, with the exception of CPS1, which showed significantly elevated levels at both day 4 and day 7 of RCCS culture (Figure 8B).

PCR analysis further demonstrated that all five urea cycle genes were upregulated after 1 and 4 days, except for ASS1, which exhibited downregulation (Figure 8C).

These results collectively indicate that RCCS effectively induces the expression of urea cycle enzymes and promotes urea synthesis in differentiated HLSCs, suggesting that these cells may contribute to the detoxification of ammonia and the maintenance of nitrogen balance.We used HepG2 and human hepatocytes (Hep) as positive control of urea cycle enzymes (Supplementary Figures S7A–C).

### 3.7 ICG uptake by hepatocyte-like HLSCs differentiated in RCCS

**FIGURE 9:**
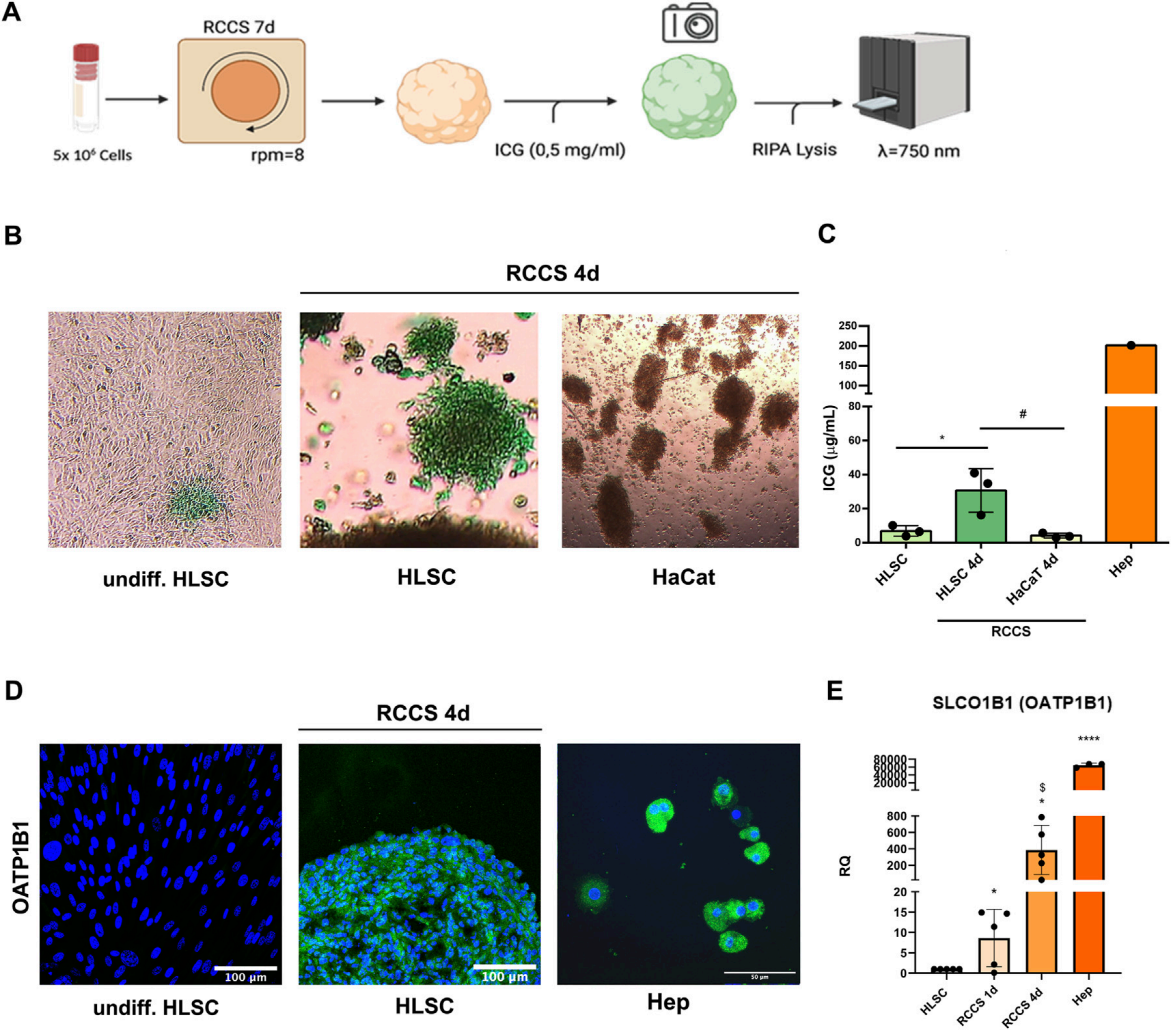
Indocyanine green (ICG) update assay. **(A)** Schematic representation of the assay. **(B)** Representative micrographs of undifferentiated, differentiated HLSCs and HaCat showing uptake of ICG after 1 h × 4 magnification. **(C)** ICG quantification in undifferentiated HLSCs, HLSCs, HaCaT and human hepatocyte (Hep) after 4 days in RCCS. Data were measured at 750 nm optical density. *p < 0.05, HLSCs RCCS 4d vs. undifferentiated HLSCs; ^#^ *p* < 0.05 HLSCs RCCS 4d vs*.* HaCaT RCCS 4d. **(D)** Representative immunofluorescence micrograph of OATP1B1 in HLSCs, RCCS differentiated HLSCs and human hepatocyte (Hep) at day 4. Human hepatocytes were used as positive control. Nuclei are stained in blue with DAPI. Scale bar, 50 and 100 µm, ×40 magnification. **(E)** Real time PCR data of OATP1B1 (SLCO1B1) in HLSC, HLSC differentiated after 1 and 4 ays, and human hepatocyte (Hep). Results were normalized using TBP and undifferentiated HLSCs were used as experimental control. Data are expressed as Relative Quantification (RQ) ±SD of five independent experiments. *p < 0.05, RCCS 1d and RCCS 4d vs. HLSC; ^$^ *p* < 0.05 RCCS 1d vs. RCCS 4d,****p < 0.0001 Hep vs. HLSC.

Indocyanine green (ICG) is an anionic dye employed to assess liver function (Ho et al., 2012). We established an *in vitro* assay to evaluate ICG uptake by differentiated HLSC aggregates (Figure 9A). As depicted in Figures 9B,C, ICG uptake was significantly higher in HLSCs after day 4 of differentiation compared to undifferentiated HLSCs and HaCaT cell aggregates (negative control).

ICG uptake is mediated by the organic anion transporting polypeptide (OATP) transporter, typically associated with mature hepatocytes (Kalliokoski and Niemi, 2009). To assess the contribution of OATP1B1 expression in HLSC differentiation, we performed immunofluorescence and real-time PCR analyses of OATP1B1 in HLSC cell aggregates. OATP1B1 expression was significantly upregulated after 1 day of differentiation and further increased after 4 days compared to undifferentiated HLSCs, human hepatocyte was used as positive control (Figures 9D,E). To assess the extent of hepatic differentiation in differentiated HLSCs, we compared the mRNA expression of the canalicular organic anion transporter 1B1 (OATP1B1), a marker of mature hepatocytes, to that of human hepatocytes. Our analysis revealed that OATP1B1 mRNA expression in differentiated HLSCs was 20-fold lower than that in human hepatocytes (Figure 9E). This finding suggests that differentiated HLSCs have been partially committed into mature hepatocyte phenotype.

## 4 Discussion

In this study, we investigated the efficiency of a 3D *in vitro* culture system to differentiate HLSCs into hepatocyte-like cells. We assessed the effectiveness of the differentiation based on the expression of hepatocyte-specific markers and on functional similarities.

Research and protocol development for stem cells differentiation into hepatocytes has been extensively studied over the past decade and more (Saito et al., 2021). The differentiation process proceeds from stem cells to hepatocyte-like cells, with an intermediate stage known as hepatoblast-like cells. Various factors have been identified that guide stem cells to differentiate into hepatocyte-like cells, typically through a stepwise strategy in 2D or 3D (Cotovio and Fernandes, 2020). Monolayer culture is the most common method used to induce differentiation, but primary human hepatocytes rapidly lose their liver-specific functions after a few days under 2D monolayer culture conditions (Elkayam et al., 2006; Kim and Rajagopalan, 2010). Additionally, a tridimensional architecture has been shown to be essential for both the endocrine and exocrine functions of the liver during organogenesis (Si-Tayeb et al., 2010).

Stem cells cultured in 3D systems, such as embryoid bodies, spheroids or scaffolds, have been reported to differentiate more efficiently into hepatocyte-like cells than stem cells cultured in 2D (Miki et al., 2011; Gieseck et al., 2014; McKee and Chaudhry, 2017). In addition to a 3D cell culture environment, the right combination of mediators and growth factors is also essential for stem cell differentiation. For example, Ogawa et al., showed that combining a 3D cell culture with a cocktail of mediators and factors, such as cAMP, BMP4, ascorbic acid, HGF, dexamethasone and oncostatin M, directed the maturation of hESCs into hESC-derived hepatocyte-like cells (Ogawa et al., 2013).

HLSCs have shown to differentiate into hepatocyte-like cells in previous studies (Herrera et al., 2006; Fonsato et al., 2010; Herrera et al., 2013; Navarro-Tableros et al., 2015). For example, HLSCs cultured in a rotary bioartificial liver device secreted HGF, albumin and urea at levels similar to hepatocytes (Fonsato et al., 2010). In another model of differentiation using decellularized liver scaffold, HLSCs upregulated the expression of lactate dehydrogenase and three subtypes of cytochrome P450. Urea nitrogen production was also confirmed in the supernatant obtained from these cultures, suggesting that HLSCs had acquired a metabolic activity similar to hepatocytes (Navarro-Tableros et al., 2015).

A significant advantage of this straightforward and time-efficient 3D method lies in its ability to characterize differentiated HLSC using a minimal number of cells (5 × 10^6^), a substantial reduction compared to the large quantities required for bioartificial liver devise (300 × 10^6^) (Fonsato et al., 2010) and liver scaffold recellularization (80–100 × 10^6^) (Navarro-Tableros et al., 2015) methods in 4 days.

In this study, we combined several methods to better characterize the differentiation capabilities of HLSCs and determine the optimal time frame for analyzing different parameters. At day 7 of culture, we observed a high percentage of apoptotic cells, especially in the core of the cell aggregates. This could be due to nutrient exhaustion and/or lack of oxygen supply (especially to the core of the aggregates) within the rotary culture vessel as the number of days of differentiation increased.

During the differentiation of stem cells from the initiation phase to the endoderm phase and finally into hepatocyte-like cells (HLCs), various stage-specific markers are expressed that can be tested (Saito et al., 2021). Additionally, the completion of the differentiation process is confirmed by specific gene expression analyses and *in vitro* functional tests (Saito et al., 2021). In this study, we showed that differentiated HLSC in respect to undifferentiated HLSC, significantly upregulated typical mature hepatocyte gene markers after differentiation, including several cytochrome P450 isoenzymes (CYP1A1, CYP1A2, CYP1B1, CYP2B6, CYP3A4, CYP3A7, and CYP7A1), two enzymes that are part of a major excretion pathway for endobiotic and xenobiotic compounds (UGT1A3 and UGT1A4), four enzymes of the urea cycle (CPS1, OTC, ARG1, and ASL), the organic anion-transporter polypeptide of the OATP family involved in the hepatic uptake of several drugs (OATP1B1), SERPINA1, FVIII, FIX and FXI. In addition, we also observed an increased gene expression of transcription factors involved in liver embryological development, such as four members of HNF family and CEBPB. Based on the membrane expression of OATP1B1 in differentiated HLSCs, we developed a functional quantitative *in vitro* assay measuring the uptake of ICG post differentiation. Additionally, functionality was also demonstrated by the secretion of urea and FVIII into the supernatant after differentiation.

Mature hepatocytes are pivotal in the coagulation cascade, synthesizing and secreting coagulation factor VIII (FVIII), a crucial protein for blood clotting (Pradhan-Sundd et al., 2021). In 2019, Pettinato et al., 2019 showed the coagulation capabilities of hepatic-like organoids derived from coculturing iPS and endothelial cells through the secretion of FVIII and FX into the conditional media peaking after 6 days of differentiation. In our 3D model, the secretion capabilities of hepatic committed HLSC were observed soon after 1 day and remain during differentiation up to 4 days. Interestingly, in the present study we showed that HLSC produced two cleavage versions of FVIII into the supernatant, mirroring the mature FVIII expression in human hepatocytes.

In undifferentiated HLSCs, the core pluripotency markers Nanog, OCT3/4, SOX2, and KLF4 were expressed. Following 3D differentiation, we observed a shift in the subcellular localization of Nanog and OCT3/4. Consistent with previous reports, Nanog, a mechanosensitive factor, undergoes translocation during the transition from cell renewal to differentiation (Gu et al., 2012; Topal et al., 2019). Given the continuous movement inherent in our 3D model, we speculate that a similar phenomenon may be occurring in our system. Additionally, we observed a transient upregulation of these markers during differentiation, followed by a subsequent downregulation. Low oxygen availability has been shown to promote the expression of Nanog, SOX2, and OCT3/4 (Mathieu et al., 2013; Tsang et al., 2013; Ahmed et al., 2016). Thus, we hypothesize that the reduced oxygen levels experienced by HLSCs within the differentiated aggregates may have contributed to the observed transient upregulation of these pluripotency markers.

ICG *in vitro* assay is a commonly used method for evaluating liver functionality (Ho et al., 2012). This assay is based on the ability of liver cells to clear ICG from circulation and excrete it into the bile. This clearance is mediated by the hepatocyte-specific transporter OATP1B1, making it an ideal assay for testing the hepatocyte-like properties of differentiated HLSCs. Human hepatocytes have a distinct pattern of ICG uptake and release that can be quantified; hepatocytes take up ICG in 30 min and then excrete the unchanged dye in 1–2 h (Ho et al., 2012). In our assay, we quantified the amount of ICG uptake by differentiated HLSCs and correlated it to the expression of OATP1B1, confirming their hepatocyte-like functional similarity.

In this study, we showed that HLSCs upregulated the expression of a wide spectrum of hepatic markers and functions from day 1 to day 4 of differentiation. These included liver-specific gene expression, urea cycle enzymes, cytochrome P450, transmembrane transporters, and coagulation factors. Additionally, *in vitro* hepatocyte-like functional activity of HLSC aggregates was confirmed by secretion of urea and FVIII and the uptake of ICG, which is mediated by the modulation of the expression of an anionic transporter in HLSC after differentiation.

This new study comprehensively characterized an *in vitro* 3D system to establish its suitability as a potency assay for qualifying the final GMP cell therapy product for HLSC, in conjunction with urea quantification or indocyanine green assay. We propose a matrix approach that combines multiple methods to characterize the differentiation status of HLSC aggregates, from which a quantitative validation assay will be selected in the future. Furthermore, our method is rapid and straightforward to implement from a GMP perspective, facilitating its transfer to a GMP setting and promoting standardization.

Taking into consideration the use of HLSCs in the GMP facility, the methods, assays, and data reported in this paper could be implemented in the validation and/or quality approval of HLSCs as a GMP final product by an official GMP-approved cell factory.

## Data Availability

The original contributions presented in the study are included in the article/Supplementary Material, further inquiries can be directed to the corresponding authors.

## Abbreviations

HLSCs, Human liver stem cells; WCB, Working cell bank; RCCS, Rotary cell culture system; ICG, Indocyanine green; HLCs, Hepatocyte-like cells; GMP, Good Manufacturing Practice.
