# Supplementary material for: In vitro characterization of 3D culture-based differentiation of human liver stem cells
Source: Front Cell Dev Biol. 2024 Feb 8;12:1352013. doi: 10.3389/fcell.2024.1352013 (PMC10881830; doi:10.3389/fcell.2024.1352013)
Supplement: Supplementary file 1 [file DataSheet1.pdf]

Supplementary Material

In vitro characterization of 3D culture-based differentiation of human liver stem cells

Marta Tapparo[1,2*], Gabriele Saccu[2,3], Chiara Pasquino[2,4], Valentina Fonsato[2,4], Claudio Medana[3], Valentina Schiavo[3], Enrica Mecarelli[3], Monica Maccagno[2,3], Lorenzo Silengo[2], Stefania Bruno[1], Giovanni Camussi[1], Maria Beatriz Herrera Sanchez[2,5*]

*** Correspondence:** Dr. Marta Tapparo, marta.tapparo@unito.it, Dr. Maria Beatriz Herrera Sanchez, mariabeatriz.herrera@unito.it

Supplementary Figures and Tables

Supplementary Table 1. Specific Primers pairs used

Gene name	Forward primer (5'-3')	Reverse primer (5'-3')
h-SLCO1B1	ACTGATTCTCGATGGGTTGGAG	TGTTTCCAGCACATGCAAAGAC
h-NANOG	ACAACTGGCCGAAGAATAGCA	GGTTCCCAGTCGGGTTCA
h-OCT4	AGCAGGAGTCGGGGTGG	AGAGCTTTGATGTCCTGGGACT
h-SOX2	TGCGAGCGCTGCACAT	GCAGCGTGTACTTATCCTTCTTCA

h-KLF4	CCATAACCAAGAGCTCATGCC	GGGCCACGATCGTCTTCC
h-TBP	TGTGCACAGGAGCCAAGAGT	ATTTTCTTGCTGCCAGTCTGG
h-FVIII	CTGTGGACGCAAGATTTCCTC	AGGATGGGAAGCCATGTTCTT
h-FIX	AGTCCTGTGAACCAGCAGTGC	GGGTGCTTTGAGTGATGTTATCC
h-FXI	CATCCCAGTGTTCTGCCATTC	AGGTAAAAAACTGGCAGCGGA

Supplementary Table 2. List of antibodies used

**Antibody**	**Catalogue number**
CD44	Miltenyi, 130-113-342
CD105	Miltenyi, 130-112-321
CD73	BD pharmingen, 560847
CD90	Miltenyi, 130-117-684
CD29	Miltenyi, 130-101-256
CD14	Miltenyi, 130-110-577
CD34	BD pharmingen, 555821
CD45	BD pharmingen, 555482
human Albumin-FITC	LSBIO, LS–C68850
human Albumin-FITC- isotype	LSBIO, LS-C149360
PE -isotype	Miltenyi, 130-113-200
FITC-isotype	Miltenyi, 130-113-199
APC-isotype	Miltenyi, 130-113-196
PCNA	Santa Cruz, sc-56
OCT3/4	R&D, MAB1759

NANOG	R&D, AF1997
SOX2	R&D, MAB2018
KLF4	R&D, AF3640
HNF4a	Abcam, 92378
ALBUMIN	R&D, MAB1455
Cytokeratin 18	Abcam, ab52948
OATP1B1/OATP2	Novusbio, NB100-74481
CYP1A1	Santa Cruz, sc-25304
CYP3A4	Santa Cruz, sc-53850
CYP7A1	Santa Cruz, sc-518007
OTC	Abcam, Ab262864
ASS1	Abcam, Ab124465<br>Abcam, Ab170952
CPS1	Abcam, Ab37168<br>Abcam, Ab45956
ARG1	Abcam, Ab239731
ASL	Abcam, Ab201025<br>Abcam, ab97370
FVIII	Abcam, ab236284<br>Abcam, ab171825
FIX	Abcam, ab236279
FXI	Abcam, ab97346
CK8	Abcam, ab59400
alpha Fetoprotein	R&D, MAB1368
GAPDH	Abcam, Ab37168

Vinculin	Self-made, kindly provided by Prof. Turco (MBC, Torino)
mouse IgG2a (negative control for OTC).	Abcam, ab18415
rabbit IgG polyclonal (negative control for ASS1)	Abcam, ab17273
rabbit IgG polyclonal (negative control for CPS1 and AGL)	Abcam, ab171870
mouse IgG2b (negative control for ARG)	Abcam, ab18469
Alexa Fluor 488 goat anti-rabbit IgG	Invitrogen, A11008
Alexa Fluor 546 goat anti-mouse IgG	Invitrogen, A11003
Alexa Fluor 594 chicken anti-goat IgG	Invitrogen, A21468
Alexa Fluor 488 goat anti-rat IgG	Invitrogen, A11006
Alexa Fluor 488 donkey anti-goat igG	Invitrogen, A11055

Supplementary Table 3. List of genes analyzed in PCR array panel.

Refseq	Symbol	Description	Name
NM_000463	UGT1A1	UDP glucuronosyltransferase 1 family, polypeptide A1	BILIQTL1/GNT1/HUG-BR1/UDPGT/UDPGT 1-1/UGT1/UGT1A
NM_019093	UGT1A3	UDP glucuronosyltransferase 1 family, polypeptide A3	UDPGT/UDPGT 1-3/UGT-1C/UGT1-03/UGT1.3/UGT1A3S/UGT1C
NM_007120	UGT1A4	UDP glucuronosyltransferase 1 family, polypeptide A4	HUG-BR2/UDPGT/UDPGT 1-4/UGT-1D/UGT1-04/UGT1.4/UGT1A4S/UGT1D
NM_002701	POUF5F1	POU class 5 homeobox 1	OCT3/OCT4/OTF-3/OTF3/OTF4/Oct-3/Oct-4
NM_022454	SOX17	SRY (sex determining region Y)-box 17	VUR3
NM_021784	FOXA2	Forkhead box A2	HNF3B/TCF3B

NM_002273	KRT8	Keratin 8	CARD2/CK-8/CK8/CYK8/K2C8/K8/KO
NM_000224	KRT18	Keratin 18	CK-18/CYK18/K18
NM_002276	KRT19	Keratin 19	CK19/K19/K1CS
NM_004364	CEBPA	CCAAT/enhancer binding protein (C/EBP), alpha	C/EBP-alpha/CEBP
NM_005194	CEBPB	CCAAT/enhancer binding protein (C/EBP), beta	C/EBP-beta/IL6DBP/NF-IL6/TCF5
NM_000545	HNF1A	HNF1 homeobox A	HNF-1A/HNF1/IDDM20/LFB1/MODY3/TCF-1/TCF1
NM_000458	HNF1B	HNF1 homeobox B	FJHN/HNF-1-beta/HNF-1B/HNF1beta/HNF2/HPC11/LF-B3/LFB3/MODY5/TCF-2/TCF2/VHNF1
NM_178849	HNF4A	Hepatocyte nuclear factor 4, alpha	FRTS4/HNF4/HNF4a7/HNF4a8/HNF4a9/HNF4alpha/MODY/MODY1/NR2A1/NR2A21/TCF/TCF14
NM_004498	ONECUT	One cut homeobox 1	HNF-6/HNF6/HNF6A
NM_000295	SERPINA1	Serpin peptidase inhibitor, clade A (alpha-1 antiproteinase, antitrypsin), member 1	A1A/A1AT/AAT/PI/PI1/PRO2275/alpha1AT
NM_000477	ALB	Albumin	ANALBA/FDAH/PRO0883/PRO0903/PRO1341
NM_001134	AFP	Alpha-fetoprotein	AFPD/FETA/HPAFP
NM_000277	PAH	Phenylalanine hydroxylase	PH/PKU/PKU1
NM_000499	CYP1A1	Cytochrome P450, family 1, subfamily A, polypeptide 1	AHH/AHRR/CP11/CYP1/P1-450/P450-C/P450DX
NM_000761	CYP1A2	Cytochrome P450, family 1, subfamily A, polypeptide 2	CP12/P3-450/P450(PA)
NM_000104	CYP1B1	Cytochrome P450, family 1, subfamily B, polypeptide 1	CP1B/CYPIB1/GLC3A/P4501B1
NM_000767	CYP2B6	Cytochrome P450, family 2, subfamily B, polypeptide 6	CPB6/CYP2B/CYP2B7/CYP2B7P/CYPIIB6/EFVM/IIB1/P450

NM_000771	CYP2C9	Cytochrome P450, family 2, subfamily C, polypeptide 9	CPC9/CYP2C/CYP2C10/CYPIIC9/P450IIC9
NM_017460	CYP3A4	Cytochrome P450, family 3, subfamily A, polypeptide 4	CP33/CP34/CYP3A/CYP3A3/CYPIIIA3/CYPIIIA4/HLP/NF-25/P450C3/P450PCN1
NM_000765	CYP3A7	Cytochrome P450, family 3, subfamily A, polypeptide 7	CP37/CYPIIIA7/P-450(HFL33)/P-450111A7/P450-HFLA
NM_000780	CYP7A1	Cytochrome P450, family 7, subfamily A, polypeptide 1	CP7A/CYP7/CYPVII
NM_001875	CPS1	Carbamoyl-phosphate synthetase 1, mitochondrial	CPSASE1/PHN
NM_000050	ASS1	Argininosuccinate synthase 1	ASS/CTLN1
NM_000531	OTC	Ornithine carbamoyltransferase	OCTD
NM_000045	ARG1	Arginase, liver	-
NM_000048	ASL	Argininosuccinate lyase	ASAL
NM_001101	ACTB	Actin, beta	BRWS1/PS1TP5BP1
NM_004048	B2M	Beta-2-microglobulin	-
NM_000194	HPRT1	Hypoxanthine phosphoribosyltransferase 1	HGPRT/HPRT
NM_002046	GAPDH	Glyceraldehyde-3-phosphate dehydrogenase	G3PD/GAPD/HEL-S-162eP
NM_000181	GUSB	Glucuronidase, beta	BG/MPS7
SA_00104	RTC	Reverse Transcription Control	RTC
	PPC	Inter Plate Reproducibility Control	PPC
SA_00105	HGDC	Human Genomic DNA Contamination	HIGX1A

Supplementary Table 4: Total RNA quantification

Sample ID	Nucleic Acid (ng/µl)
HLSC sample 1	452.4
HLSC sample 2	478.7
HLSC sample 3	471.6
HLSC sample 4	847.3
HLSC sample 5	856.9
RCCS 1d sample 1	636.1
RCCS 1d sample 2	672.2
RCCS 1d sample 3	586.2
RCCS 1d sample 4	1576.7
RCCS 1d sample 5	996.3
RCCS 4d sample 1	536.6
RCCS 4d sample 2	387.7
RCCS 4d sample 3	134.8
RCCS 4d sample 4	431.6
RCCS 4d sample 5	360.4
RCCS 7d sample 1	70.0
RCCS 7d sample 2	75.5
RCCS 7d sample 3	70.9

Supplementary Table 5. PCR array gene expression

Genes	Symbol	Fold Change (comparing to HLSC group)								
		RCCS 1d			RCCS 4d			Hepatocyte (Hep)		
		Fold Change	Note	p-value	Fold Change	Note	p-value	Fold Change	Note	p-value
1	UGT1A1	0.80	**B**	0.8495	1.09	**B**	0.5704	**1580.24**	**A**	**0.0420**
2	UGT1A3	1.48	**B**	0.0797	**3.15**	**B**	0.1945	**4775.16**	**A**	0.1432
3	UGT1A4	**1.67**	**B**	0.07318	**2.41**	**B**	0.1520	**9846.08**	**A**	0.0603
4	POUF5F1	**2.38**		**0.0338**	**5.34**		**0.0000**	**10.04**		0.2164
5	SOX17	**0.20**		**0.0014**	**0.44**		**0.0089**	**0.61**	**A**	0.3324
6	FOXA2	1.36	**B**	0.2658	**2.11**		**0.0429**	**4750.95**	**A**	0.1648
7	KRT8	**1.53**		0.1919	**2.71**		**0.0039**	**6.29**		0.2149
8	KRT18	**0.08**		0.0606	**0.22**		0.0820	1.02		0.7571
9	KRT19	**0.13**	**A**	**0.0400**	**0.08**	**A**	**0.0349**	**3.87**	**A**	0.2240
10	CEBPA	1.41	**B**	0.9481	**1.50**	**B**	0.8788	**4564.16**	**A**	**0.0091**
11	CEBPB	0.86		0.4240	**1.55**		0.3872	0.82		0.5550
12	HNF1A	**1.76**	**B**	0.1776	**3.08**	**B**	0.0845	**861.91**	**A**	0.1901
13	HNF1B	1.47		**0.0214**	**3.49**		**0.0443**	**529.20**	**A**	0.192
14	HNF4A	**1.61**	**B**	0.1784	**2.12**		**0.0284**	**1045.85**	**A**	**0.0486**
15	ONECUT	**2.87**		**0.0236**	**8.68**		**0.0009**	**3840.47**	**A**	**0.0339**
16	SERPINA1	**2.44**		**0.0157**	**8.74**		**0.0108**	**192646.9**	**A**	0.1425
17	ALB	**1.78**		**0.0447**	**2.17**	**B**	0.064	**298878.5**	**A**	0.1983
18	AFP	1.19	**B**	0.6400	0.78	**B**	0.4402	**54.44**	**B**	0.2115

19	PAH	1.38	**B**	0.1786	**3.28**	**B**	0.1705	**13016.0**	**A**	0.1503
20	CYP1A1	**16.25**	**A**	**0.0101**	**35.10**	**A**	0.0548	**528.95**	**A**	0.1542
21	CYP1A2	1.10	**C**	0.426	**2.11**		**0.0228**	**6028.85**	**A**	0.0987
22	CYP1B1	**9.00**		**0.0054**	**25.76**		**0.0000**	**10.22**		**0.0512**
23	CYP2B6	1.10	**C**	0.4260	**2.12**		**0.0354**	**2026.82**	**A**	**0.0193**
24	CYP2C9	1.10	**C**	0.4260	1.79	**C**	**0.0016**	**21400.15**	**A**	0.1702
25	CYP3A4	**3.86**		**0.0110**	**11.36**		**0.0113**	**9564.01**	**A**	0.1776
26	CYP3A7	**2.96**		**0.0019**	**5.85**		**0.0000**	**86.69**	**B**	0.0833
27	CYP7A1	1.14	**B**	0.7137	**2.70**		**0.0416**	**384.37**	**A**	0.0881
28	CPS1	**2.03**		**0.0000**	**0.70**		**0.0003**	**19.72**		0.174
29	ASS1	**0.49**		**0.0018**	1.54		**0.0045**	**13.89**		**0.0567**
30	OTC	1.22	**B**	0.2275	**2.37**	**B**	0.0799	**5361.17**	**A**	0.1569
31	ARG1	**1.72**		0.0595	**2.20**		**0.0007**	**278.29**		0.1354
32	ASL	1.45		0.0561	**2.15**		**0.0022**	**9.24**		**0.0328**
33	ACTB	**0.55**		**0.0325**	**0.33**		**0.006**	**0.55**		0.8272

Supplementary Table 5. PCR array gene expression analysis. Data are presented as fold-change ($2^{-\Delta\Delta CT}$), which is the normalized gene expression ($2^{-\Delta CT}$) in the test sample in respect to the normalized gene expression ($2^{-\Delta CT}$) in the control sample. p values are calculated using a student's t-test of the replicate $2^{-\Delta CT}$ values for each gene in the control group and treatment groups. p values less than 0.05 are indicated in blue.

Supplementary Table 5 Legend:

A: If a gene's average threshold cycle (Ct) value is relatively high (>30) in either the control or test sample, and reasonably low (<30) in the other sample, this suggests that the gene's expression is relatively low in one sample and reasonably detected in the other sample. In this case, the actual fold change value is likely to be at least as large as the calculated and reported fold change result. However, the fold change result may also have greater variation if the p-value is greater than 0.05.

B: If a gene's average threshold cycle (Ct) value is relatively high (>30) in both the control and test samples, and the p-value for the fold change is either unavailable or relatively high (p > 0.05), this suggests that the gene's expression is low in both samples. However, it is also possible that the gene is expressed in one sample but at a level below the limit of detection. In this case, the fold change result may not be accurate and may have greater variation.

C: If a gene's average threshold cycle (Ct) value is either not determined or greater than the defined cut-off (default 35) in both samples, this means that its expression was undetected. As a result, the fold change result for this gene is erroneous and uninterpretable.

Supplementary Table 6: Total Protein quantification

Sample ID	Protein (mg/mL)
HLSC sample 6	2.61
HLSC sample 7	2.89
HLSC sample 8	2.08
RCCS 4d sample 6	2.61
RCCS 4d sample 7	3.32
RCCS 4d sample 8	1.94
RCCS 7d sample 6	1.48
RCCS 7d sample 7	1.36
RCCS 7d sample 8	1.33

Supplementary Figure 1.

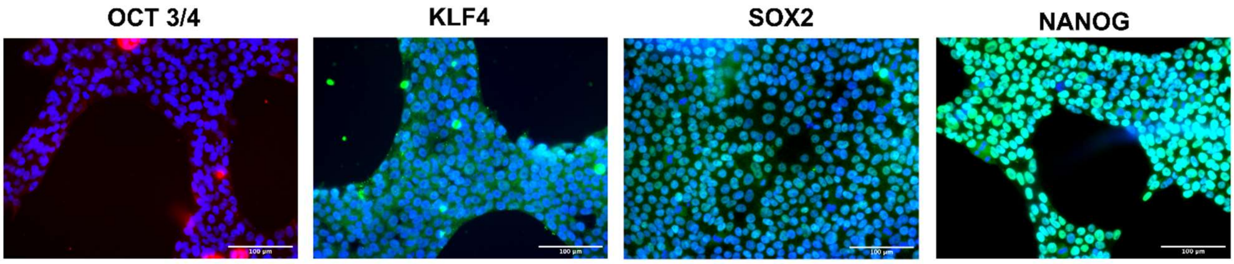

Supplementary Figure 1. Stem cell markers on H9 (embryonic stem cell line; positive control). Representative immunofluorescence micrographs of OCT3/4, KLF4, SOX2 and NANOG in H9. Nuclei were stained in blue with DAPI. Scale bar, 100µm.

Supplementary Material

Supplementary Figure 2.

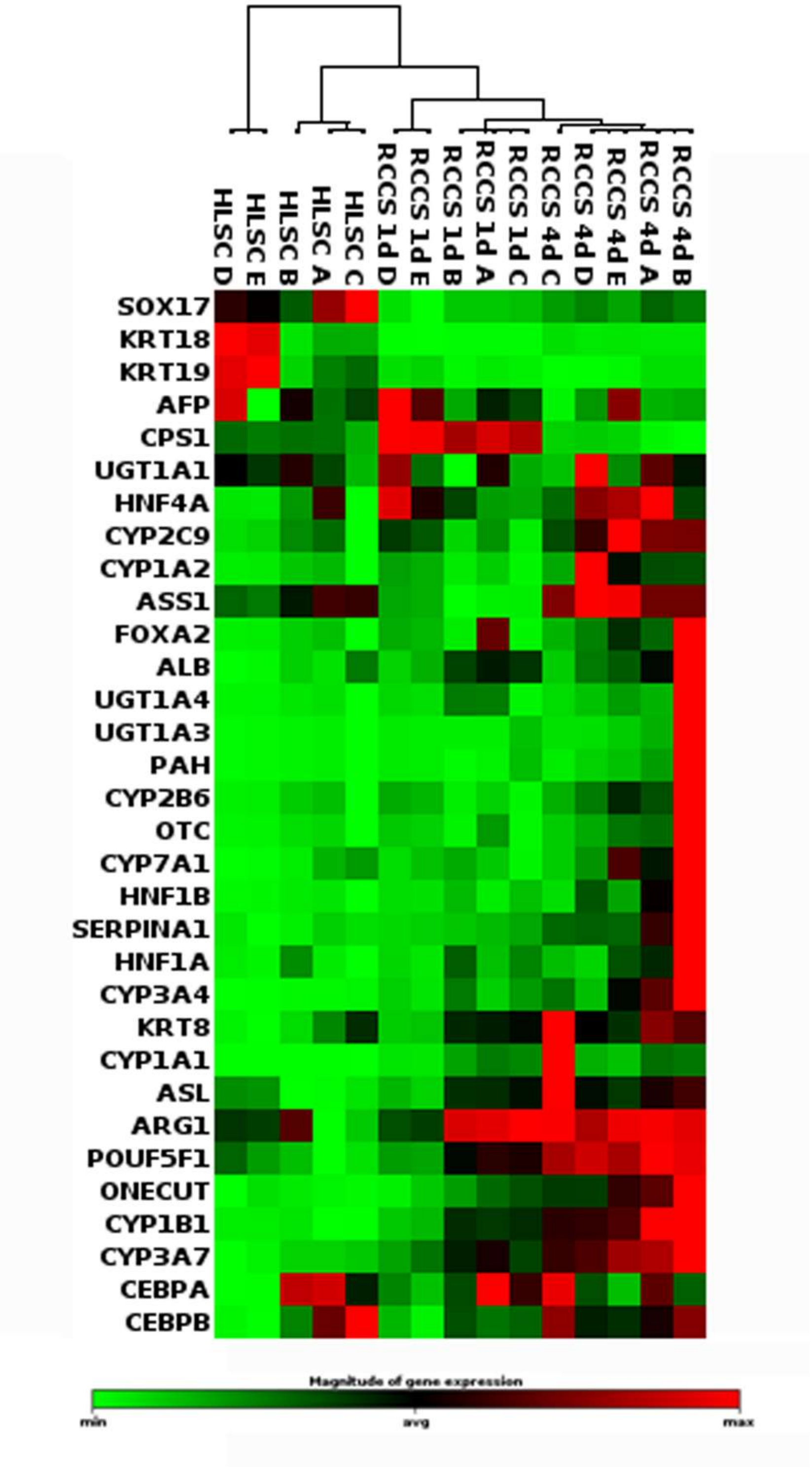

Supplementary Figure 2. Heatmap of PCR array gene expression. Heatmap of single sample gene expression of five biological groups analyzed.

Supplementary Figure 3.

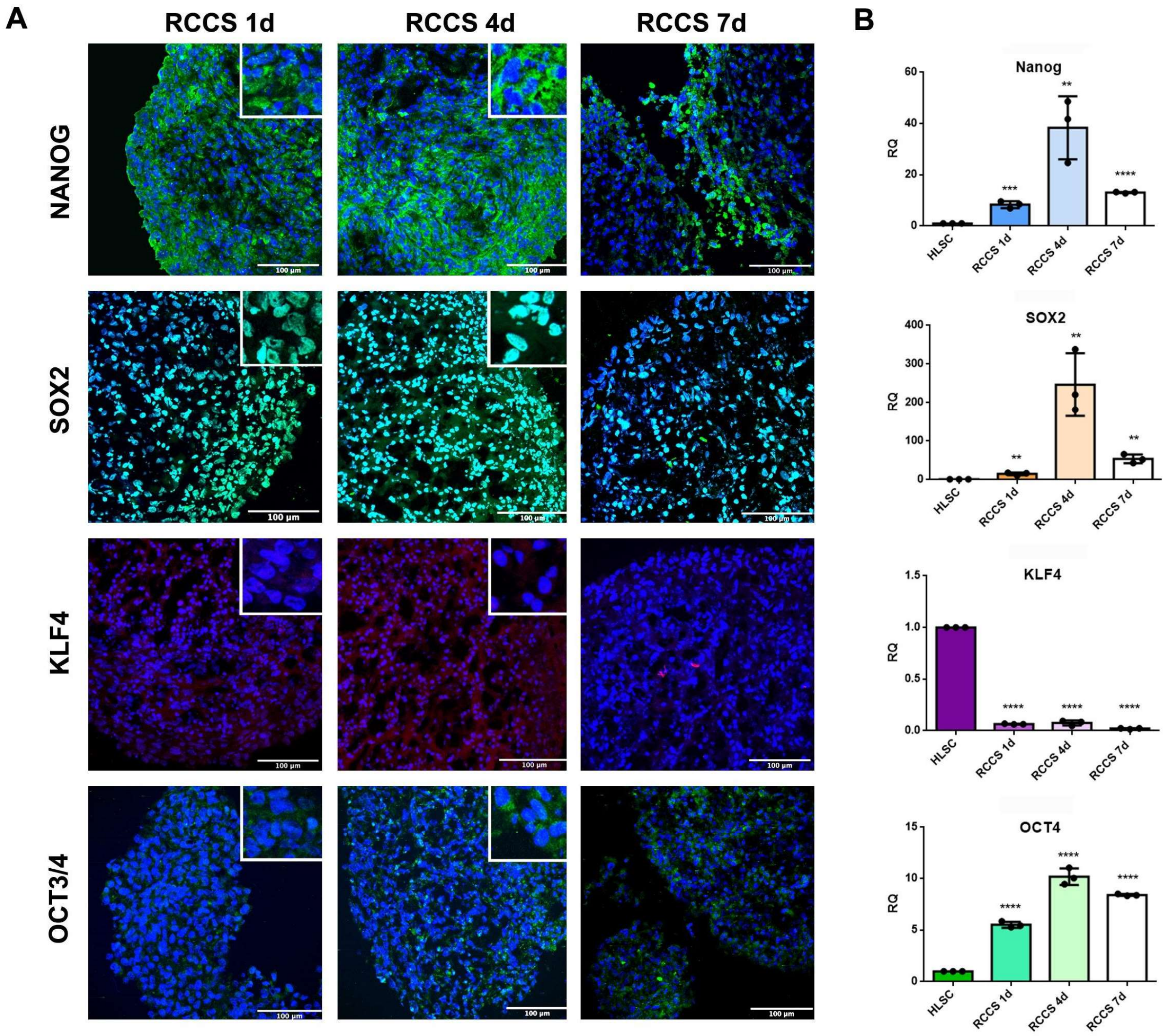

Supplementary Figure 3. Stem cell markers. (A) Representative immunofluorescence micrograph of stem cell markers Nanog, SOX2, KLF4, OCT3/4 in undifferentiated HLSCs and RCCS differentiated HLSCs. Scale bar, 100µm; magnification 40X, insert pictures. Nuclei are stained in blue with DAPI. **(B)** Real-time PCR of the embryonic stem cells genes. Results were normalized using TBP and undifferentiated HLSCs were used as control. Results are expressed as Relative Quantification (RQ). The results are presented as mean values of five independent experiments ±SD. * p<0.05, ** p<0.01, *** p<0.001, **** p<0.0001 RCCS 1d and 4d *vs.* HLSC.

Supplementary Figure 4.

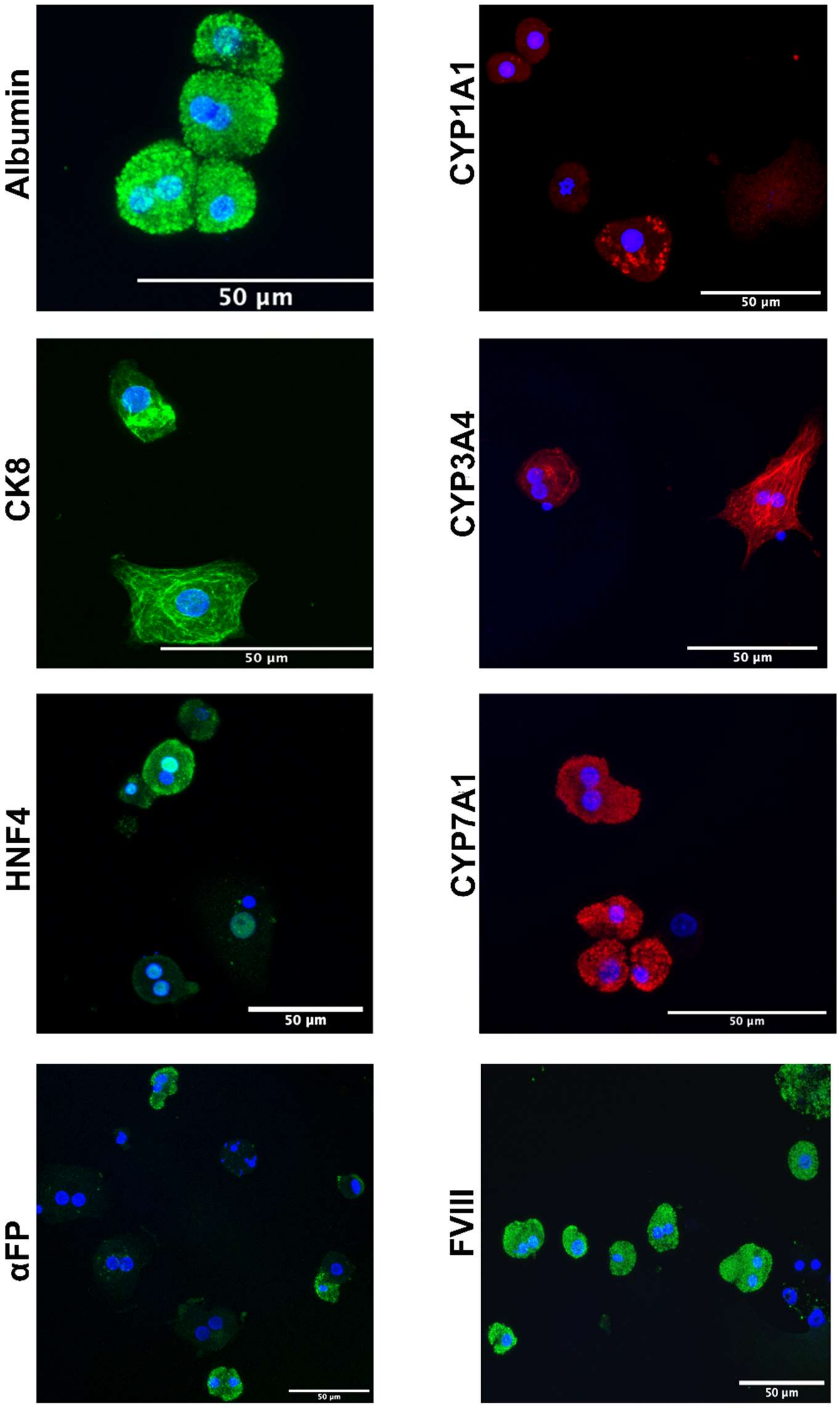

Supplementary Figure 4. Hepatic markers. Representative immunofluorescence micrograph data of hepatic markers in human hepatocytes. Scale bar 50µm. Nuclei were stained in blue with DAPI.

Supplementary Figure 5.

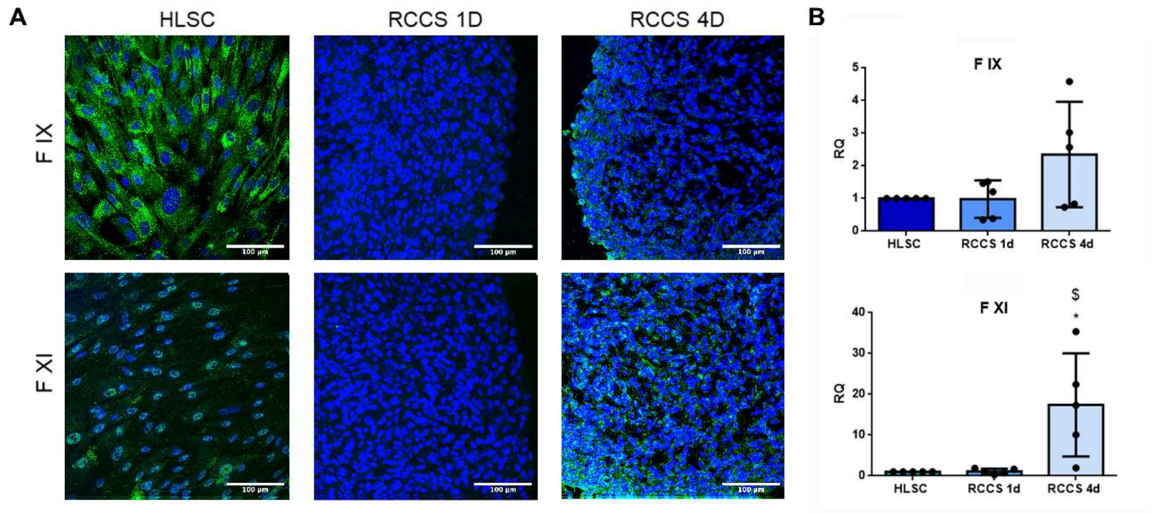

Supplementary Figure 5. Coagulation Factor expression. (**A**)Representative immunofluorescence micrograph data of coagulation factor IX (FIX) and coagulation factor XI (FXI) in HLSC and HLSC aggregates after differentiation in RCCS. Scale bar 100µm. Nuclei were stained in blue with DAPI. (**B**) Quantitative real-time PCR analysis showed that the expression of coagulation factor genes was upregulated in differentiated HLSC compared to undifferentiated HLSC. Results are normalized to TBP and expressed as relative quantification (RQ). Data are presented as mean values of five independent experiments ±SD. * $p<0.05$ RCCS 4d vs. HLSC; $ $p<0.05$ RCCS 4d vs RCCS 1d.

Supplementary Figure 6.

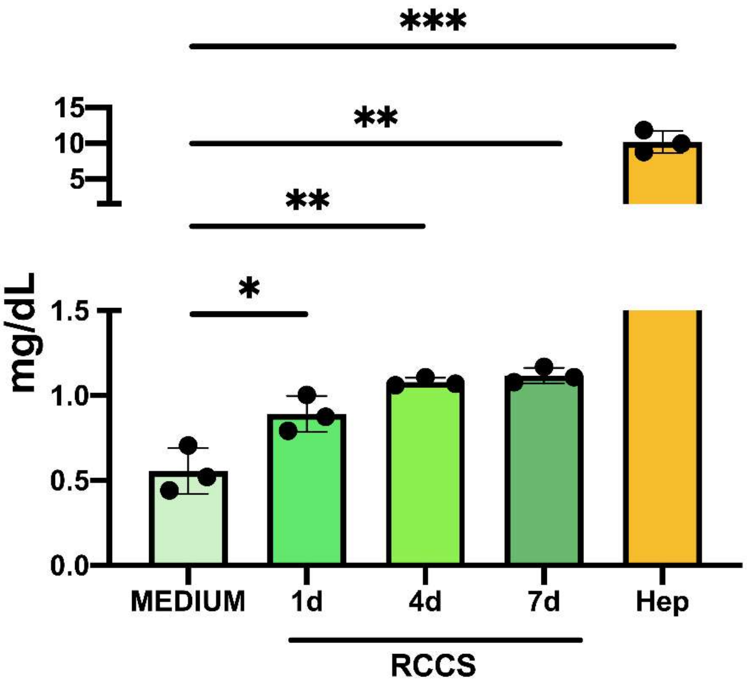

Supplementary Figure 6. Urea secretion. Urea into the supernatant of differentiated HLSC in RCCS after 1, 4, 7 days and human hepatocytes after 4 days in RCCS was quantified by Quantichrom assay kit. Results were expressed as mg/dL of urea. The results are presented as mean values obtained from three independent experiments ±SD * p<0.05, ** p<0.01, *** p<0.001 HLSC cultured in RCCS 1d, 4d, 7d and human hepatocyte (Hep) *vs.* medium along.

Supplementary Figure 7.

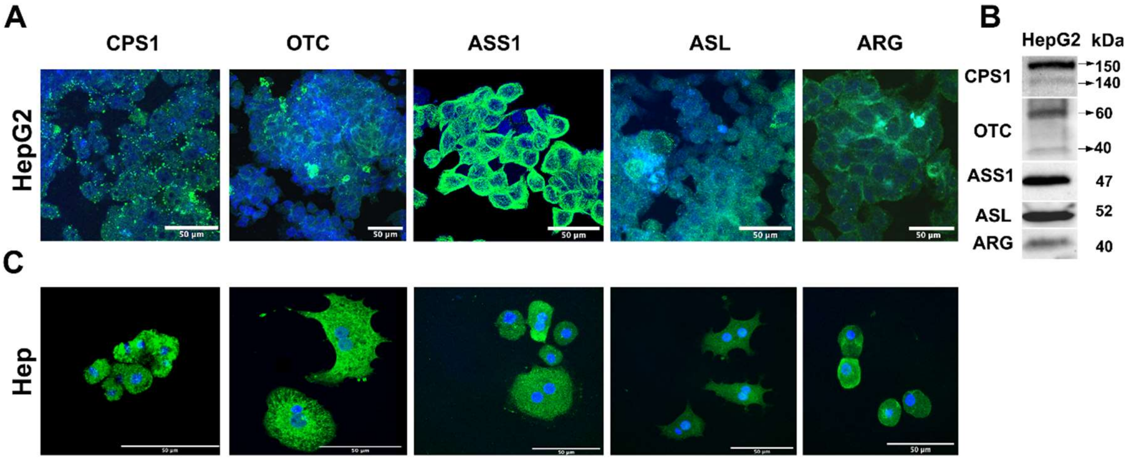

Supplementary Figure 7. Urea cycle enzyme on HepG2 (positive control). (**A**) Representative immunofluorescence micrographs of urea cycle enzymes CPS1, OTC, ASS1, ASL and ARG in HepG2. Nuclei were stained in blue with DAPI. Scale bar, 50 µm. (**B**) Representative western blot of urea cycle enzymes in HepG2 cells used as positive control. (**C**) Representative immunofluorescence micrographs of urea cycle enzymes CPS1, OTC, ASS1, ASL and ARG in human hepatocytes (Hep). Nuclei were stained in blue with DAPI. Scale bar, 50 µm.

Supplementary Figure 8.

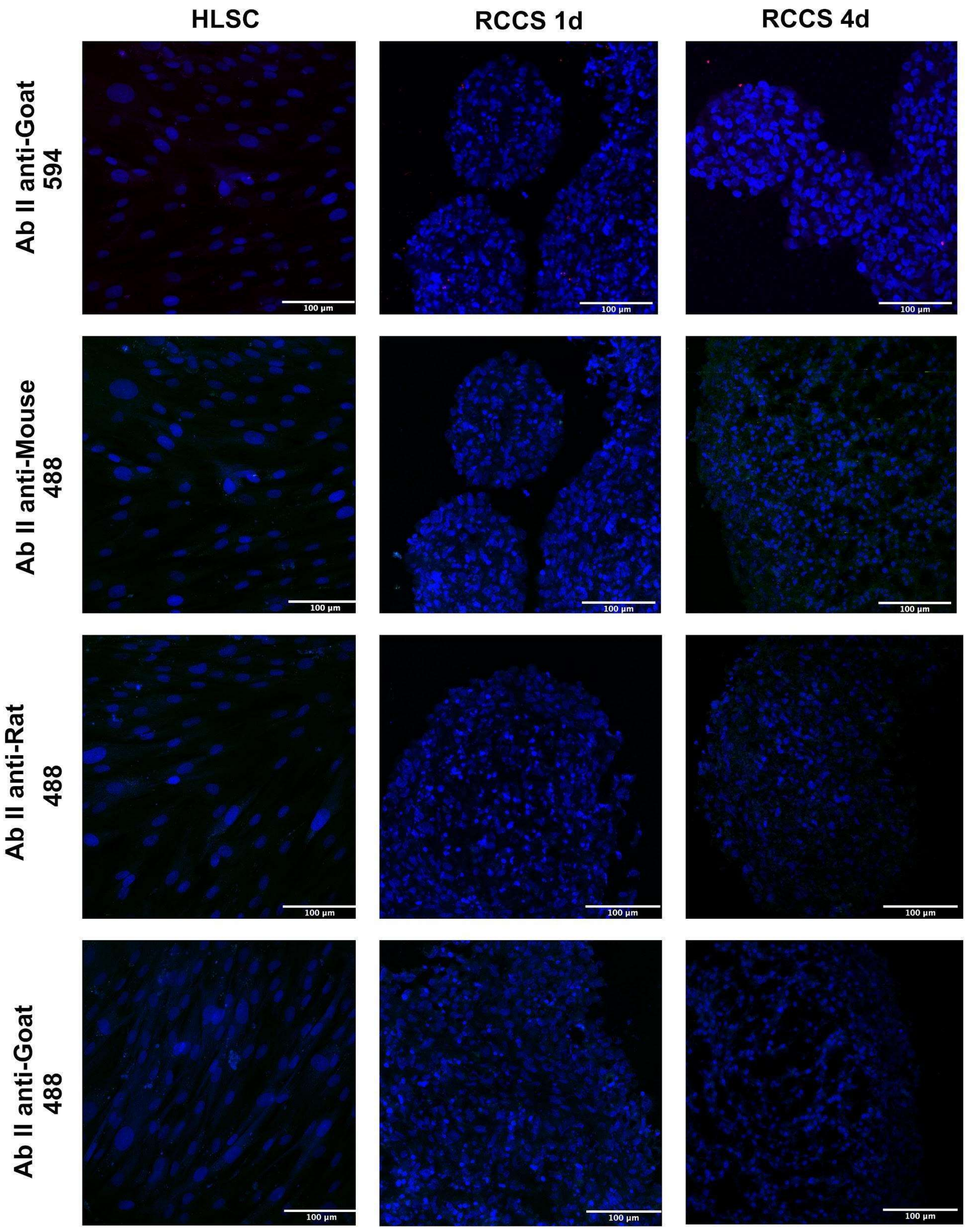

Supplementary Figure 8. Immunofluorescence analysis of negative controls. Representative immunofluorescence micrograph of secondary antibodies used to characterize HLSC differentiation. Secondary antibodies: anti-Goat 594 nm (red staining), anti-mouse, anti-rat, anti-goat 488 (green staining). Nuclei were stained in blue with DAPI. Scale bar: 100 µm.
